# Implications of glycosylation for the development of selected cytokines and their derivatives for medical use

**DOI:** 10.1016/j.biotechadv.2024.108467

**Published:** 2024-10-22

**Authors:** Giulia Scapin, Ece Cagdas, Lise Marie Grav, Nathan E Lewis, Steffen Goletz, Lise Hafkenscheid

**Affiliations:** a Department of Biotechnology and Biomedicine, Mammalian Cell Line Engineering, Technical University of Denmark, Søltofts Plads, 2800 Kgs Lyngby, Denmark; b The Novo Nordisk Foundation Center for Biosustainability, Technical University of Denmark, Søltofts Plads, 2800 Kgs Lyngby, Denmark; c Department of Pediatrics, University of California, San Diego, La Jolla, CA, USA; d Department of Bioengineering, University of California, San Diego, La Jolla, CA, USA; e Department of Biotechnology and Biomedicine, Biotherapeutic Glycoengineering and Immunology, Technical University of Denmark, Søltofts Plads, 2800 Kgs Lyngby, Denmark

**Keywords:** Glycosylation, Cytokines, Engineering, Therapeutics, Cancer, Auto-immunity, Immunocytokines

## Abstract

Cytokines are important regulators of immune responses, making them attractive targets for autoimmune diseases and cancer therapeutics. Yet, the significance of cytokine glycosylation remains underestimated. Many cytokines carry *N*- and *O*-glycans and some even undergo *C*-mannosylation. Recombinant cytokines produced in heterologous host cells may lack glycans or exhibit a different glycosylation pattern such as varying levels of galactosylation, sialylation, fucosylation or xylose addition compared to their human counterparts, potentially impacting critical immune interactions.

We focused on cytokines that are currently utilized or designed in advanced therapeutic formats, including immunocytokines, fusokines, engager cytokines, and genetically engineered ‘supercytokines.’ Despite the innovative designs of these cytokine derivatives, their glycosylation patterns have not been extensively studied. By examining the glycosylation of the human native cytokines, G-CSF and GM-CSF, interferons β and γ, TNF-α and interleukins-2, −3 −4, −6, −7, −9, −12, −13, −15, −17A, −21, and − 22, we aim to assess its potential impact on their therapeutic derivatives. Understanding the glycosylation of the native cytokines could provide critical insights into the safety, efficacy, and functionality of these next-generation cytokine therapies, affecting factors such as stability, bioactivity, antigenicity, and half-life. This knowledge can guide the choice of optimal expression hosts for production and advance the development of effective cytokine-based therapeutics and synthetic immunology drugs.

## 1. Introduction

Cytokines are small signaling molecules that range from 6 to 70 kDa and are produced by various cell types, primarily neutrophils, monocytes, macrophages, T and B cells (Zhang and An, 2007). They are critical players in our immune system to amplify or dampen responses against environmental threats such as bacteria, viruses, and other pathogens. Cytokines are categorized into different classes including growth factors, interferons (IFNs), tumor necrosis factors (TNFs) and interleukins (ILs) (Thomson and Lotze, 2003). They are defined as pleiotropic, as they interact with specific receptors on the surfaces of different cell types. In addition, cytokines also exhibit functional redundancy, indicating that multiple cytokines can perform similar biological activities. For instance, both IL-2 and IL-15 can induce natural killer (NK) and T-cell proliferation (Becknell and Caligiuri, 2005).

Different cytokines are involved in numerous biological processes such as innate immunity, adaptive immunity, wound healing, and tolerance induction. Hence, cytokines exhibit both proinflammatory and anti-inflammatory functions: while proinflammatory cytokines (*e.g.*, TNFα, IL-6) gather and stimulate immune cells, anti-inflammatory cytokines (*e.g.*, IL-4, IL-10) facilitate tissue healing by suppressing inflammation. Therefore, imbalances in cytokine homeostasis and signaling can lead to different complications from autoimmunity to cancer. This dual nature makes them both attractive targets and tools in various therapies (Pires et al., 2021).

In autoimmunity and inflammatory diseases, several biologics have been designed to regain balance by reducing the ongoing proinflammatory cytokine responses. Examples of these drugs include adalimumab (anti-TNF), tocilizumab (anti-IL-6), and anakinra (anti-IL1β) to treat rheumatoid arthritis and Crohn’s disease. *Vice versa*, in cancer treatment, the stimulation of pro-inflammatory processes is desired. Cytokines, such as IL-2 and IFNα, were identified as the first immunotherapeutic molecules due to their ability to trigger pro-inflammatory processes (Qu et al., 2018). Both were initially approved by the Food and Drug Administration (FDA) as a monotherapy. However, the low response rate and the high toxicity associated with high-dose administration, have relegated their clinical use (Berraondo et al., 2019). To overcome these severe side effects, more targeted approaches have emerged where anti-cancer antibody treatments, inducing processes like Fc-mediated Antibody-Dependent Cell-mediated Cytotoxicity (ADCC) and Complement-Dependent Cytotoxicity (CDC), are combined with cytokines, both as adjuvants or fusion constructs in different formats. In these therapies, the added effect of cytokines is leveraged to initiate specific immune responses that help eradicate the tumor (Holder et al., 2022).

**Fig. 1.**
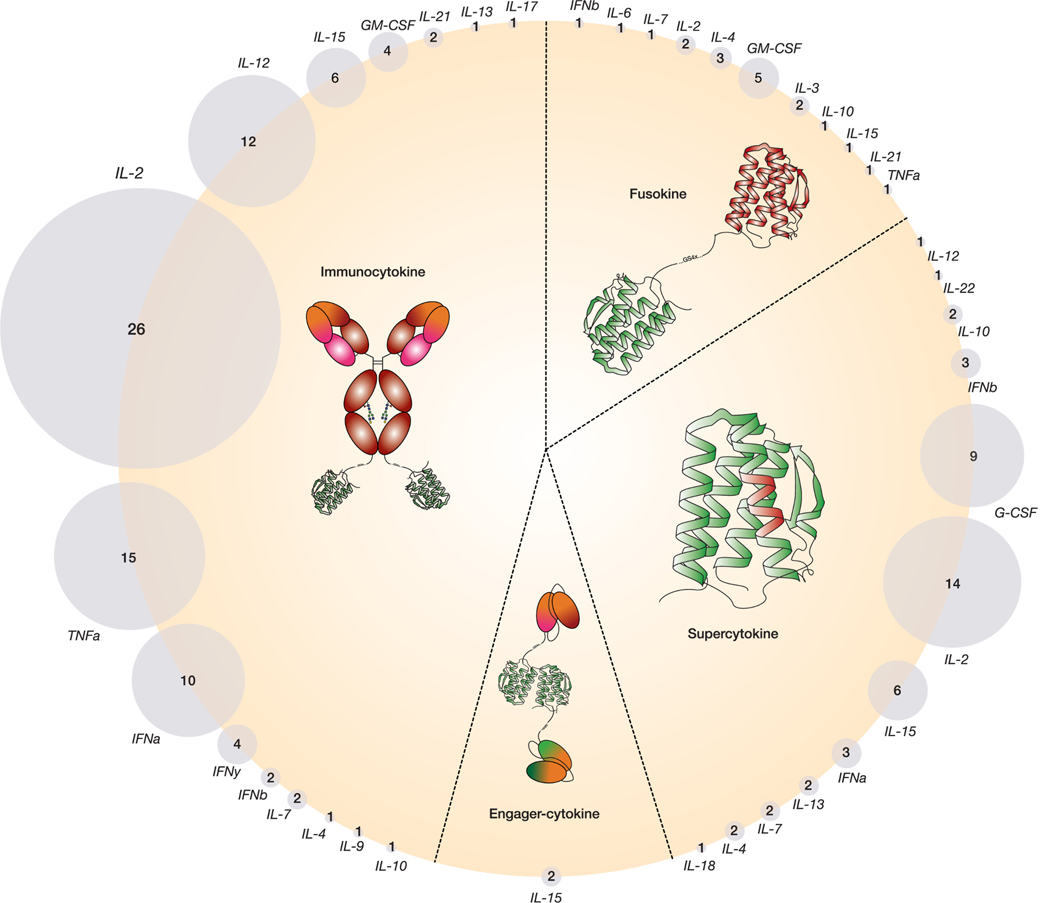
Therapeutic cytokines derivatives in the form of immunocytokines, supercytokines, engager cytokines and fusokines. Cytokines have been functionalized in various ways for therapeutic applications. These include: supercytokines (designed to enhance their functional properties, such as increased potency or extended half-life), immunocytokines (cytokine fusion proteins for targeted delivery to specific cells or tissues), engager cytokines (cytokine combinations with two targeting components to bring together effector functions), and fusokines (multi-cytokine fusions that harness their synergistic effects). Presented here are cytokine fusions and formats developed from 2019 to 2022, with the gray circles next to each cytokine indicating the number of therapeutic cytokine formats or fusions reported in the literature and recent reviews (Table 1).

Enthusiasm for cytokine engineering has sparked increased research interest in the development of novel cytokine-based therapies, driving the introduction of a plethora of new terms such as supercytokines, immunocytokines, engager cytokines, fusokines, neoleukins, and synthekines (Fig. 1). Supercytokines are cytokines with enhanced therapeutic potential from binding domain modifications to increase receptor affinity/specificity, to point mutations or PEGylation (addition of polyethyleneglycol) to increase their half-lives, or fusion with new protein domains (*e.g.*, antibody Fc, albumin, IL15Rα, *etc.*). Meanwhile, immunocytokines are therapeutic antibodies that are “armed” with cytokines, ultimately aiming to boost tumor clearance. Similarly, engager cytokines are cytokines linked with two different single-chain variable fragments (scFvs) (*e.g.*, BiTE, BiKE) which bind to antigens in both cancer and T or NK cells, triggering increased targeted tumor killing. Lastly, fusokines are chimeric cytokines created by combining two different cytokines. Additional classes of engineered cytokine therapies include neoleukins, *in silico*-designed mimetic cytokines, and synthekines, cytokine fusion variants with different receptor-subunit affinity (Zheng et al., 2022). In the future, new engineering strategies will further improve cytokine half-life and specificity, while reducing toxicity.

Adding to the intricate nature of cytokine signaling, many cytokines undergo glycosylation with covalent attachment of mono or oligosaccharides. Glycosylation is one of the most abundant co/posttranslational modifications, and since their structures emerge through complex biosynthetic pathways involving over 150 different glycosyltransferases, a diverse array of glycoforms can emerge with varying properties that influence glycoprotein function. Glycosylation contributes to protein diversification, potentially affecting their biological activity, antigenicity, stability, diffusibility, tissue distribution, pharmacokinetics (*in vivo* and *in vitro* half-lives), and target cell interactions (Schjoldager et al., 2020).

**Fig. 2.**
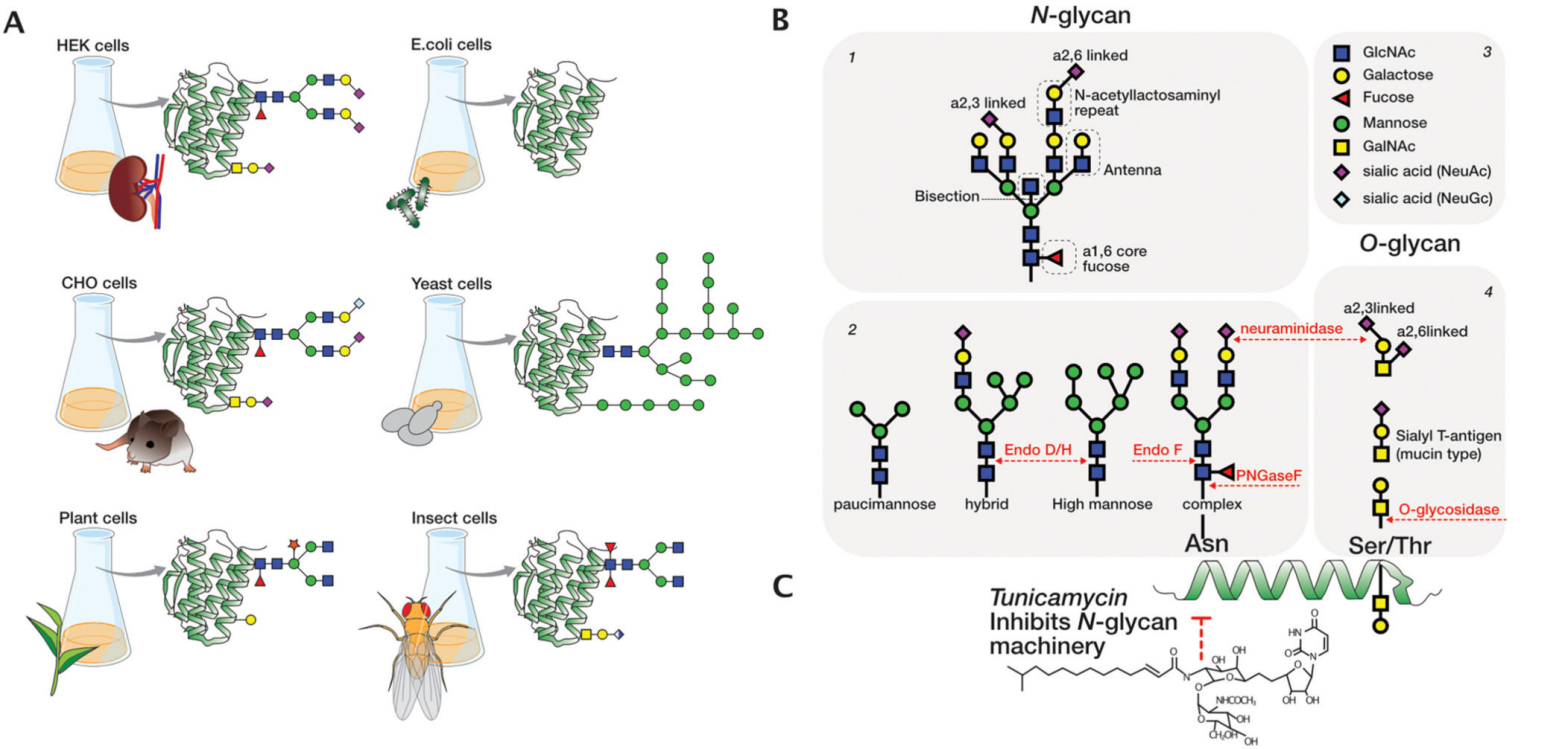
Comprehensive overview of glycan structures, glycosidases and glycan inhibitors, and potential implications in protein expression systems on the glycosylation phenotype. A) Different host expression systems can be used for protein production, but each presents different glycoprofiles, which can potentially impact the functions of a protein of interest. Depicted are representations of the most commonly observed glycophenotypes in the different host systems, where occupancy or glycan type can change upon different growth conditions and genetic modifications.B) Nomenclature for Glycans: (1/4) *N*-glycans can be classified into hybrid, high mannose, complex types and paucimannose. *N*-glycans attach at asparagine residues (N-X(=∕P)-S/T), while *O*-glycans are added to serines or threonines. A common *O*-glycan in this review is the mucin-type, which consists of GalNAc, galactose, and sialic acids. Glycans can also be branched and further extended with a GlcNAc-galactose unit, referred to as a LacNAc repeat. Finally, both *O*-glycans and *N*-glycans can be capped with sialic acid in α2,6 or α2,3 linkages, or include fucose often linked to GlcNAc per galactose. (2) Glycosidases cut glycans and they are used to release glycans from proteins for glycan profiling, glycophenotypes validation or functional studies. For example, PNGase F cleaves between asparagine (Asn) and the first GlcNAc, while Endo F cleaves between the two GlcNAc residues. Endo D and H also cleave between GlcNAc residues but only in high mannose, paucimannose, and hybrid structures. Sialidases are particularly used to confirm the presence of sialic acid or for removal to study the functional effect of sialic acid on the proteins (3) Common monosaccharides in mammalian *N*-/*O*-glycans are shown. C) Tunicamycin can be used to hinder the transfer of UDP-GlcNAc to dolichol phosphate, resulting in the complete inhibition of *N*-glycosylation machinery. It has been employed to investigate the functional implications of *N*-glycans on cytokines.

The glycosylation patterns of cytokines can impact their functional properties and roles in immune responses. Many cytokines exhibit different *N*-, *O*- or *C*-glycoforms, comprising both glycosylated and non-glycosylated species when isolated from human tissues. This inherent heterogeneity underscores the importance of understanding the role of cytokine glycosylation, starting with their early investigation in the 1990s. However, challenges in isolating cytokines in sufficient quantities from natural sources prompted the exploration of alternative expression systems, such as *Escherichia coli* (*E. coli*), which lacks the *N*-, *O*-, and *C*-glycosylation machinery. Yeasts, insect cells, plants, and mammalian cells are also used for cytokine production, but each heterologous expression platform provides variations in the glycosylation patterns of the recombinant cytokines, which can have either a significant or neutral effect on their biological activity (Fig. 2A)(Das et al., 2022).

**Fig. 3.**
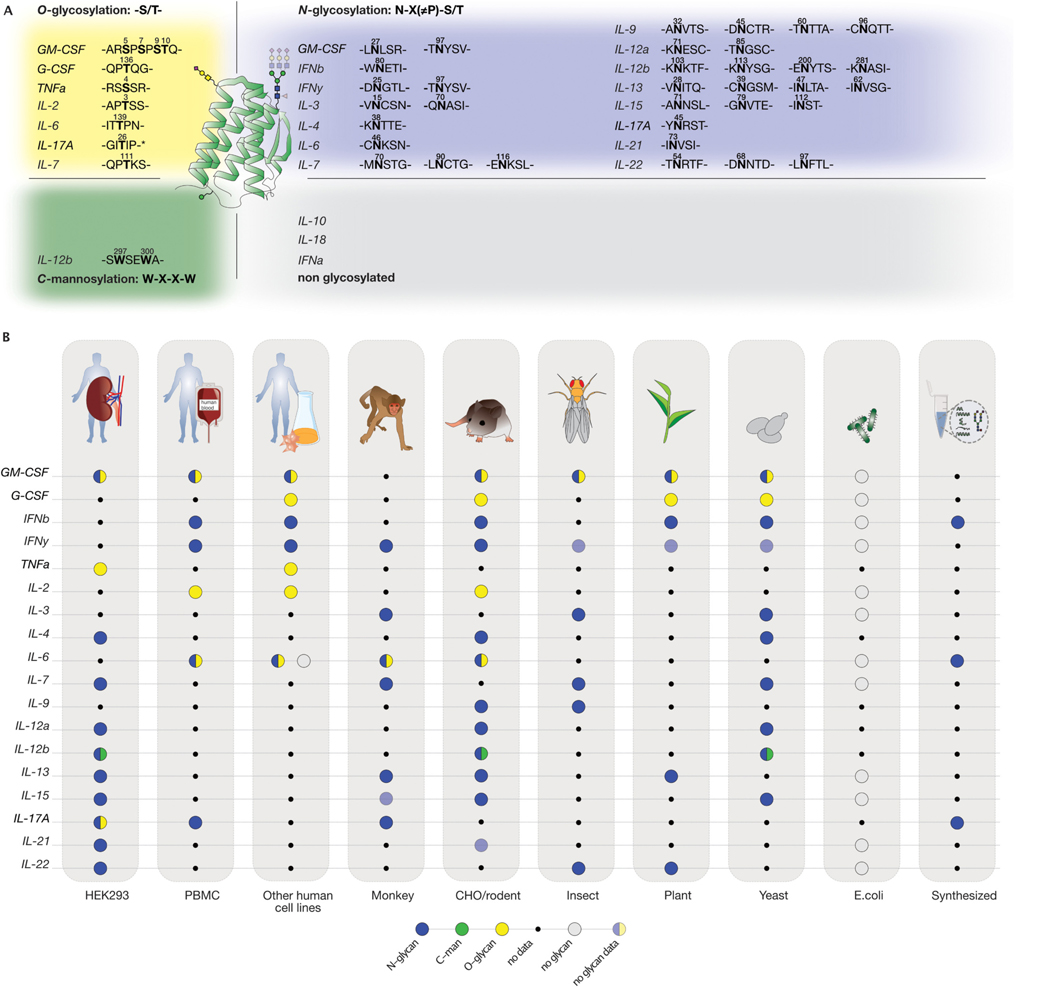
Major glycosylation sites for example cytokines. A) Glycan sites have been identified and validated for many cytokines, including example shown here. *O*-glycan sites are represented by a yellow background on the top left of the figure, while *N*-glycan sites are indicated with a blue background on the top right. *C*-mannosylation sites are shown with a green background on the bottom left, and non-glycosylated cytokines are presented in gray. All glycan sites are denoted in bold, accompanied by the amino acid number after cleavage of the signal peptide, as indicated by UniProt. In the case of G-CSF the glycan site refers to the longer isoform A (isoform B, Thr133). For IL-17A, a star symbol denotes an alternative *O*-glycan site observed exclusively in recombinant IL-17A. B) Expression of each example cytokine has been reported in a variety of host cell lines or semi-synthetic systems. The presence of *N*-glycosylation on each protein is represented by a blue circle, *C*-mannosylation by a green circle, and *O*-glycosylation by a yellow one. A gray circle indicates that the host cell does not glycosylate the cytokine. A small dot signifies that the host was not used for expressing or studying the cytokine. When the circle is transparent, it indicates that the host was reported to be used for expressing the cytokine of interest, but there was no data on the glycosylation profile. Additional details on the production cell line used can be found in Table 2.

**Fig. 4.**
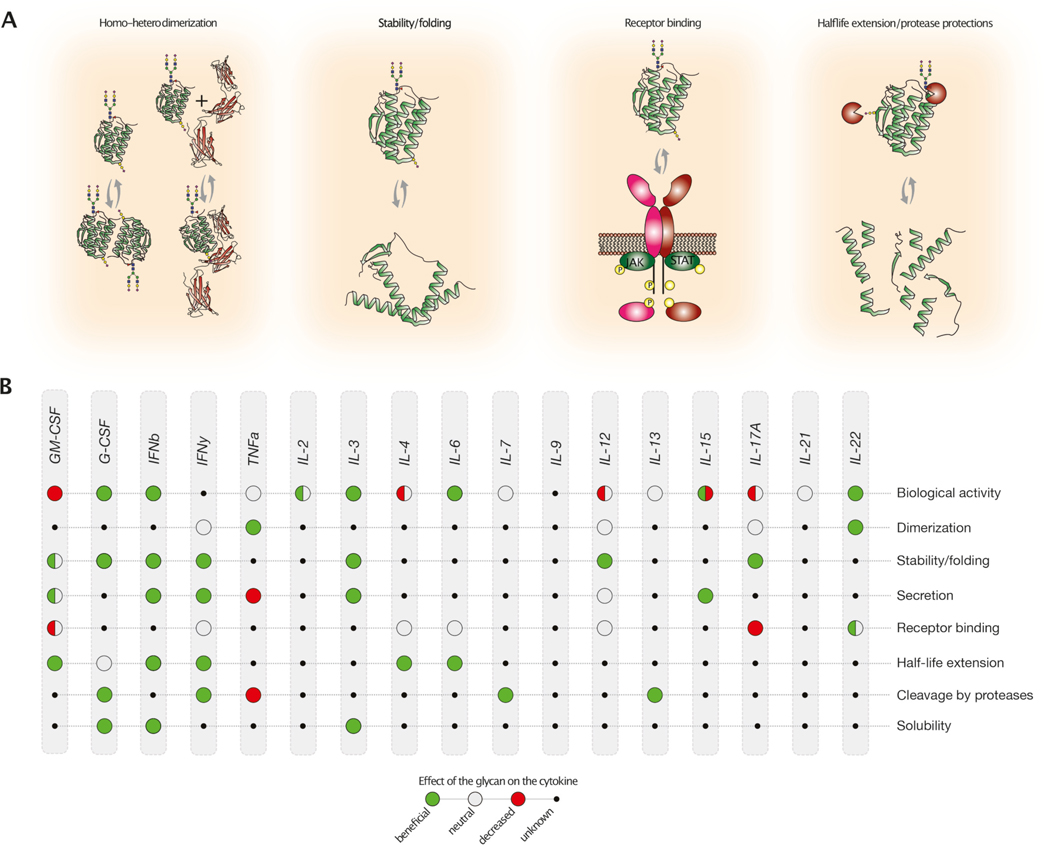
Effect of glycans on cytokine function. A) Glycans can have various effects on cytokine function. B) The reported effects of glycosylation on cytokine functions can be categorized into various aspects: overall biological activity, homo-heterodimerization, stability/folding, secretion, receptor binding, half-life extension, and vulnerability to protease decay. A green circle indicates if a beneficial effect of glycans has been reported on the cytokine’s function, while a red circle indicates a negative effect. A gray circle represents no observed effect of glycans on the particular function, while a black circle shows where impacts on the specific function have not been reported. Lastly, a dual-colored circle indicates conflicting literature, where beneficial, negative, and/or no effects were reported for the same cytokine. This figure depicts the overall effect of glycosylation, without distinguishing between *N*-glycosylation, *O*-glycosylation, and *C*-mannosylation.

Given the rapid evolution of new cytokines engineering derivatives (Fig. 1, Table 1) and their widespread use in medical research and clinical applications, it is noteworthy that 80 % of the cytokines implemented in new therapeutic designs are glycoproteins with potential glycosylation sites based on their genetic code (Fig. 3A, Table 2) (Deckers et al., 2023; Gout et al., 2022; Zheng et al., 2022). A prior review in 1995 explored the functional importance of cytokine glycosylation (Opdenakker et al., 1995). However, there has not been a focused review since then. Here, we describe the glycosylation patterns of cytokines that have been selected for designing new cytokine fusion therapeutics. Additionally, new therapeutic formats include cytokines such as IFNα, IL-10, and IL-18, which are not glycosylated and will not be discussed further. This highlights the importance of choosing the appropriate production platforms to achieve the desired therapeutic or immunological effects. For each cytokine, and if the implemented fusion constructs are available, we describe which type of glycosylation was identified since their original isolation or production (Fig. 3, Table 2) and explore the potential impact of glycans on cytokine function when reported (Fig. 4).

### 1.1. Granulocyte macrophage colony stimulating factor

The growth factor Granulocyte-Macrophage Colony Stimulating Factor (GM-CSF) promotes the proliferation and maturation of myeloid progenitor cells and is widely used in differentiating monocytes into dendritic cells (DC). Its involvement in various diseases has been established, and GM-CSF has been a component in clinical trials (de Souza Vieira et al., 2023), and included in the development of four distinct immunocytokines for cancer therapy (Gasson, 1991; (Lotfi et al., 2019). Moreover, GM-CSF has been administered in the fusokine configuration in conjunction with IL-2, IL-4, and other cytokines (Fig. 1, Table 1) (Deng et al., 2014; Penafuerte et al., 2009).

Regarding glycosylation, two potential *N*-glycosylation sites were identified Asn27 and Asn37(Cebon et al., 1990). GM-CSF is also *O-*glycosylated on Ser5, Ser7, Ser9 and Thr10 when expressed in COS7 cells (Fig. 3, Table 2) (Kaushansky et al., 1987; Kaushansky et al., 1992). Insights from a glycan analysis approach of Chinese Hamster Ovary cells (CHO)-derived GM-CSF revealed the presence of bi-, tri-, and tetra-antennary *N*-glycans, containing 1 or 3 *N-*acetyllactosamine (LacNac) repeats within the antennae (Fig. 2B.1). Additionally, these *N*-glycans were core-fucosylated and capped with sialic acid. Notably, *N-*glycans site-specific analysis revealed variable site occupancy with Asn37 being occupied approximately at 85 % and Asn27 at 60 %. As for *O*-glycans, a mucin-type structure was confirmed (NeuNAcα2–3Galβ1–3GalNAc) (Forno et al., 2004). While Ser7, Ser9, and Thr10 were glycosylated, Ser5 displayed lower occupancy in CHO cells, compared to other mammalian cell types (Cebon et al., 1990)(Forno et al., 2004). In a complementary study, CHO-derived GM-CSF isolated from adherent cultures exhibited two occupied *N*-glycan sites whereas the suspension cultures produced GM-CSF with only one *N-*glycan site occupied. However, the GM-CSF from adherent cells were less sialylated (Forno et al., 2004). The heterogeneity of GM-CSF glycosylation pattern was further amplified by the selection of various recombinant expression platforms, including *E. coli* (Burgess et al., 1987), yeast (Miyajima et al., 1986) and other mammalian cells such as CHO (Wingfield et al., 1988), Namalwa cells (Okamoto et al., 1990) COS7 cells (Kaushansky et al., 1987)(Figs. 2A and 3B). For example, in contrast to CHO cells, activated human lymphocytes expressed predominantly GM-CSF with a single *N*-glycan (Cebon et al., 1990).

Further investigation into the extent of glycosylation on GM-CSF involved three distinct glycoforms of the protein expressed in human Namalwa cells. These glycoforms included variants with two *N*-glycan sites occupied (2 N), one *N-*glycan site glycosylated (1 N), or no *N*-linked glycosylation, while all variants were *O-*glycosylated (O). Remarkably, the 2 N + O glycoform exhibited significantly reduced activity, approximately 200-fold less than the non-glycosylated *E. coli*-derived GM-CSF. In contrast, the 1 N + O and O GM-CSF variants demonstrated intermediate activities. Similar results were observed when studying GM-CSF secreted from peripheral blood lymphocytes (PBLs), due to reduced receptor-binding interactions (Cebon et al., 1990). However, the role of *O*-glycans remains unclear, as their presence does not show significant differences in terms of receptor binding, the ability to differentiate HL-60 cells or to have an impact on neutrophil activity when compared to non-glycosylated *E.coli*-derived GM-CSF (Cebon et al., 1990). Glycans on GM-CSF may influence its secretion. Site-directed mutagenesis of all *N*- and *O-*glycosylation sites led to decreased transient secretion, compared to the recombinant *O*-glyco-sylated or fully glycosylated GM-CSF in COS cells, but in a complementary study, no differences were observed (Kaushansky et al., 1987; Kaushansky et al., 1992). Indeed, tunicamycin-treated phytohemagglutinin (PHA)-induced lymphocytes still secreted GM-CSF and the role of glycans in secretion is still uncertain (Fig. 2C) (Cebon et al., 1990). *In vivo*, Namalwa-derived GM-CSF exhibited a fivefold decrease in clearance rate from the bloodstream in rats treated with the 2 N + O GM-CSF (5 h (h)) compared to *E.coli*-derived and non-*N*-glycosylated GM-CSF (1 h). Moreover, the 2 N + O GM-CSF also had a significantly longer time to reach its maximum concentration in the blood than the non-glycosylated forms (Okamoto et al., 1991). For CHO-derived GM-CSF, *N-*glycosylation does not enhance protein stability (Wingfield et al., 1988), suggesting that other mechanisms may be involved in extending the half-life of glycosylated GM-CSF. Beyond its biological function, it is noteworthy that GM-CSF lacking glycosylation developed neutralizing antibodies in a clinical study (Gribben et al., 1990).

GM-CSF glycosylation has been extensively studied, providing a thorough overview of the relation between glycosylation status and its role in cytokine function (Fig. 4). While, fully *N-* and *O-*glycosylated GM-CSF seems to decrease cytokine biological activity, removing the *N*-glycans can instead increase it to an extent that generates a hyperactive form (Asn27His), leading to the development of extreme pathogenic reactions in patients affected by Behcet disease (Rösler et al., 2021). Furthermore, the removal of glycans exposes GM-CSF epitopes that are usually masked, triggering an immune response against new drug designs (Gribben et al., 1990). By carefully controlling the glycosylation status of GM-CSF, it may be possible to optimize its therapeutic benefits while minimizing potential risks to patients.

### 1.2. Granulocyte colony stimulating factor

Granulocyte colony-stimulating factor (G-CSF) is primarily produced by macrophages, monocytes, fibroblasts, and endothelial cells (Cetean et al., 2015). G-CSF plays a role in hematopoiesis by stimulating the differentiation of neutrophil precursors and the proliferation, and mobilization of neutrophils (Bronchud et al., 1988; Lord et al., 1989; Yamamoto et al., 1993). Recombinant G-CSF is implemented in treating neutropenia in patients undergoing chemotherapy (Bönig et al., 2001). To enhance the therapeutic efficacy, novel G-CSF supercytokines have been developed by engineering G-CSF with modifications such as glycan addition, PEGylation and fusion with fragment crystallizable (Fc) tails, albumin and albumin binding domains (ABDs) (Fig. 1, Table 1) (Alshehri et al., 2014; Molineux, 2004; Cox et al., 2004; Ghidini et al., 2016; Nikravesh et al., 2022).

Four alternatively spliced mRNA isoforms of G-CSF have been recently reported (A, B, C, and D) in different cancer cell lines (Toghraie et al., 2019). Among them, isoform A with a molecular weight (MW) of 19 kilodalton (kDa) was purified from the human G-CSF-producing tumor cell line, CHU-2 (Nomura et al., 1986). Around the same time, isoform B, with a MW of 18.4 kDa, was purified from the constitutively producing human bladder carcinoma cell line 5637 (Welte et al., 1982). The cDNAs of the major G-CSF isoforms, A and B, which differ by only three amino acids (Val-Ser-Gln) after Leu35, were later cloned in monkey COS cells and no *N*-glycosylation sites were identified (Nagata et al., 1986). The first amino acid and sugar composition analysis of G-CSF was performed on isoform A, indicating a potential *O*-linked glycan site (Nomura et al., 1986). However, isoform B demonstrated superior bioactivity and stability, making it the preferred variant for commercial recombinant production. Thus, all the subsequent references to G-CSF pertained specifically to isoform B (Gascon, 2012; Nagata et al., 1986). Glycan characterization on CHO derived-G-CSF identified the *O*-glycosylation site at Thr133 by peptide sequencing (Kubota et al., 1990), revealing an *O*-glycan containing *N*-acetylgalactosamine (GalNAc), galactose, and sialic acids (Fig. 3A, Table 2) (Oheda et al., 1988). Subsequent Nuclear Magnetic Resonance (NMR) spectroscopy determined the glycan composition as mucin type glycan (NeuAcα2–3Galβ1–3 (±NeuAcα2–6) GalNAc) (Fig. 2B.4) (Kubota et al., 1990; Oheda et al., 1988). Detailed glycan analysis on *Pichia pastoris* (*P. pastoris)*-derived G-CSF by liquid-chromatography mass spectrometry (LC-MS) and anion-exchange chromatography (HPAEC-PAD) showed that the *O*-glycan site is 55 % occupied with an *O*-mannose (Fig. 2A) (Gong et al., 2014). Moreover, in plants, G-CSF with mammalian-like glycan structure was obtained by co-expressing G-CSF with *O*-glycosylation machinery elements in *Nicotiana benthamiana* (*N. benthamiana*) (Fig. 3B) (Ramírez-Alanis et al., 2018).

Currently, two recombinant G-CSF molecules derived from *E. coli* and CHO cells are available on the market (Theyab et al., 2022). The first approved recombinant human G-CSF was generated as non-glycosylated in *E. coli* (Frampton et al., 1994; Welte et al., 1996; Yang et al., 2012) with a fast clearance rate from the bloodstream (Cooper et al., 2011; Hoggatt and Pelus, 2014; Molineux, 2004; Petros et al., 1997). *In vivo* studies demonstrated that administering the glycosylated CHO-derived G-CSF (Lenograstim) resulted in a greater ability to mobilize CD34+ cells in individuals compared to treatment with the non-glycosylated cytokine (Filgrastim) (Ataergin et al., 2008). Glycosylation on CHO-derived G-CSF may protect against protein oxidation due to the carbohydrate shielding a sulfhydryl group on a Cys17, thereby improving cytokine stability (Cooper et al., 2011; Hasegawa, 1993). Notably, compared to non-glycosylated G-CSF, the glycosylated form showed improved stability *in vitro, in vivo* serum half-life and similar clinical outcomes in patients with neutropenia (Bönig et al., 2001; Tanaka et al., 1997; Watts et al., 1997). Neutrophils can secrete elastase, which generates a negative feedback loop in granulopoiesis and inhibits G-CSF activity *in vitro* (El Ouriaghli et al., 2003). Interestingly, *E. coli* derived G-CSF was fastly degraded by elastase in a time and dose dependent manner, while the CHO-derived G-CSF was more resistant to the elastase degradation (Carter et al., 2004). Furthermore, glycosylated human (CHU-2) and CHO-derived G-CSF showed enhanced resistance to heat denaturation, reduced tendency to polymerize (Oh-eda et al., 1990), and similar *in vitro* and *in vivo* bioactivity (Fig. 4) (Kubota et al., 1990).

As previously reported, glycosylation has been implemented to extend G-CSF half-life. (Alshehri et al., 2014; Chung et al., 2011). In particular, scientists introduced a novel *N*-glycosylation site on G-CSF by site-directed mutagenesis (Phe140Asn) resulting in a G-CSF variant with enhanced ability to stimulate hematopoietic cell proliferation and differentiation compared to single *O*-glycosylated G-CSF (Chung et al., 2011). Besides mutagenesis, native G-CSF was coupled to tandem glycosylation motifs, containing two, four, or eight motifs (G-CSF2NAT, G-CSF4NAT, G-CSF8NAT). The additional verified *N*-glycans improved G-CSF biological activity and extended the serum half-life in rats compared to the non-glycosylated controls, where Asn was replaced with Glu (Alshehri et al., 2014).

G-CSF glycosylation plays a role in preventing its rapid clearance from the bloodstream. Recently, the FDA approved a G-CSF supercytokine, which consists of G-CSF fused with an Fc fragment and a PEG linker to extend its half-life (Schwartzberg et al., 2020; Theyab et al., 2022; Pechtner et al., 2024). While glycosylation enhances the protein’s *in vitro* bioactivity and stability, it does not significantly impact its clinical therapeutic efficacy (Bönig et al., 2001; Tanaka et al., 1997; Watts et al., 1997). Studies have primarily focused on enhancing cytokine bioavailability by modifying G-CSF, including *N*- or *O-*glycan addition, which prolonged its half-life compared to the non-glycosylated form (Alshehri et al., 2014; Bondarenko et al., 2013; Mahlert et al., 2013). Thus, glycoengineering of G-CSF holds great potential to maximize its therapeutic activity.

### 1.3. Interferon-β

Like interferon-alpha, interferon beta (IFN-β) is a type I interferon produced by various cell types, including plasmacytoid DC, DC, monocytes and macrophages upon viral infection. IFN-β shares the same receptor as IFN-α, the IFNAR, initiating the Jak/Stat signaling pathway (Kalliolias and Ivashkiv, 2010). IFN-β has been used to treat viral infections, including COVID-19 (Sosa et al., 2021). However, recombinant IFN-β and newly designed IFN-β derivatives, including fusion molecules with anti-albumin fragment antigen-binding region (Fab) fragments or Galectin-9, have been implemented to treat relapsing-remitting multiple sclerosis (Clerico et al., 2007; Tovey and Lallemand, 2010; Ji et al., 2019; Hamana et al., 2018) (Fig. 1, Table 1). Furthermore, IFN-β has shown anti-tumor potential, as demonstrated by its incorporation into a fusion construct with an anti-Her2 antibody (Lee et al., 2020), suggesting its broad applicability in new cytokine fusion formats for treating different diseases.

The cDNA isolated from human fibroblasts revealed that IFN-β harbors a potential glycosylation site at position Asn80 (Derynck et al., 1981; Goeddel et al., 1980) (Fig. 3A, Table 2). Immune Complex (IC) stimulated primary human fibroblasts expressed glycosylated IFN-β at Asn80, confirmed by Raman spectroscopy (Hosoi et al., 1988). Tunicamycin treatment of murine MG-63 cells (Fig. 2C) producing IFN-β revealed a MW shift from 22 to 25 kDa to 18 kDa (Watanabe and Kawade, 1983; Karpusas et al., 1997; Dissing-Olesen et al., 2008). A comparable size difference was identified between IFN-β expressed in CHO cells and IFN-β produced in *E. coli*, which lacks *N*-glycosylation capacity (Colby et al., 1984). In later studies, CHO-derived IFN-β showed 100 % occupancy of Asn80, indicating that mammalian expression systems effectively glycosylate IFN-β (Song et al., 2020). Both *S. cerevisiae* and *P. pastoris* produce glycosylated IFN-β, with *P. pastoris* achieving approximately 75 % glycosylation occupancy at Asn80 (Tomimoto et al., 2013; Skoko et al., 2003). The glycan profiling of CHO-derived IFN-β exhibited 63–95 % core-fucosylated biantennary glycans capped with α2,3-sialic acid using mass spectrometry analysis. The remaining 5–25 % of glycans were likely tri- or higher antennary structures (Conradt et al., 1987; Dissing-Olesen et al., 2008; Mastrangeli et al., 2015). A small amount of *N*-Glycolylneuraminic acid (NeuGc), a sialic acid not synthesized by humans, was also detected (Bush et al., 2016) (Fig. 2A and B.3). Besides CHO cells, IFN-β derived from PC8, C127, and IC-stimulated human fibroblasts were glycoanalyzed. Surprisingly, native IFN-β contained exclusively α2,3-sialic acid, similar to CHO-derived IFN-β, but with a lower proportion of biantennary glycans (68 %). In contrast, C127 mouse cells produced human IFN-β with exclusively α2,6-linked sialic acid. IFN-β derived from PC8 lung adenocarcinoma cells exhibited a higher percentage of triantennary glycans (30 %), bisected glycans (20 %), and low amounts of Gal1–3 capping, which is typically observed in mouse and cancer cells (Kagawa et al., 1988). Lastly, when expressed in *P. pastoris*, IFN-β exhibited high-mannose structures containing up to 18 mannose (Skoko et al., 2003). Given the variability in IFN-β glycosylation patterns across different expression systems, the World Health Organization convened a panel of experts to establish international standards for clinical IFN-β preparations (Meager and Das, 2005), leading to the use of CHO and *E. coli*-derived IFN-β as expression systems (Fig. 3B). To this end, both the *E. coli*-derived IFN-β variant named IFN-β−1b and CHO-produced IFN-β marketed as IFN-β−1a have been implemented in the treatment of relapsing-remitting multiple sclerosis.

The biochemical properties of IFN-β glycosylation in human MG-63 cells were previously analyzed after tunicamycin treatment (Fig. 2C). Glycosylated IFN-β demonstrated slightly enhanced heat resistance compared to its non-glycosylated counterpart. Furthermore, tunicamycin treatment of MG-63 cells resulted in a significant reduction in the production levels of IFN-β (Watanabe and Kawade, 1983). Similarly, in human FS-4 fibroblasts, cell treatment with glycosylation inhibitors led to a 90 % reduction in IFN-β production (Yip and Vilček, 1981; Havell et al., 1975), suggesting that glycosylation may play a crucial role in protein stability and/or secretion. NMR and circular dichroism studies revealed no significant conformational changes between the glycosylated human fibroblast-derived and non-glycosylated *E. coli*-derived IFN-β variants, indicating that glycosylation does not affect the overall protein structure (Utsumi et al., 1986). Removing glycans from CHO-expressed IFN-β using Peptide N-Glycosidase F (PNGase F) led to protein precipitation, implying a role of glycans in increasing protein solubility (Conradt et al., 1987). A crystal structure of CHO-derived IFN-β revealed surface-exposed hydrophobic residues near Asn80, indicating that glycan might shield these hydrophobic patches, enhancing protein solubility. In particular, the core 1,6-fucose moiety interacts with the side chains of Asn86 and Gln23, forming at least two internal hydrogen bonds, likely contribute to a more stable molecular structure (Karpusas et al., 1997; Karpusas et al., 1998).

In terms of biological activity, inducible IFN-β overexpressing CHO cell line produced glycosylated and low amounts of non-glycosylated IFN-β variants. The non-glycosylated form had a 300-fold decreased antiviral activity compared to the glycosylated form (McCormick et al., 1984). Similarly, *E. coli*-produced IFN-β displayed reduced activity and production challenges, including dimer and oligomer formation. Introducing a single point mutation Cys17Ser in *E. coli*-produced IFN-β yielded a properly folded protein with bioactivity similar to that of human fibroblast IFN-β (Mark et al., 1984). A direct comparison of IFN-β−1a (glycosylated produced in CHO) and IFN-β−1b (*E. coli*-produced), including PNGase F-treated IFN-β−1a, revealed a three-fold reduction in antiviral activity for the soluble fraction of the deglycosylated IFN-β−1a. Notably, IFN-β−1b displayed a ten-fold lower antiviral activity, likely due to aggregation, as determined by size-exclusion chromatography. Deglycosylation of CHO-derived IFN-β resulted in a two-fold decrease in its ability to induce B cell proliferation and a ten-fold decrease in its ability to stimulate leukocytes in a mouse model (Dissing-Olesen et al., 2008). Overall, these findings suggest that glycosylation enhances IFN-β solubility, stability, and biological activity (Runkel et al., 1998).

Investigating the role of specific glycan structures, the complete removal of sialic acids after short incubation (2 h) of IFN-β−1a with sialidases maintained its ability to induce leukocytes activation. However, a longer incubation (48 h) significantly reduced IFN-β−1a bioactivity, similar to the effect obtained with PNGaseF treated IFN-β−1a, while fucosidase treatment had no effect in the same *in vitro* assay. This suggests that sialylation may play a role in protein stability, as the proteins exhibited increased aggregation following the extended 48-h sialidase treatment (Dissing-Olesen et al., 2008). However, a subsequent study failed to replicate the reported stabilizing effect of sialic acid (Mastrangeli et al., 2015). In contrast, sialic acid removal was beneficial in treating hepatitis B virus (HBV) infection, as demonstrated in both *in vitro* and *in vivo* assays. In fact, asialofetuin administration prevented non-sialylated (asialo) IFN-β binding to the asialoglycoprotein receptors on the liver cell line and in the HBV infection mouse model, suggesting that asialo-IFN-β could be re-directed *via* asialoglycoprotein receptor to the liver, increasing the cytokine stimulated antiviral activity (Eto and Takahashi, 1999). Increasing IFN-β glycan antennarity enhanced its antiviral activity and proliferative effect by major histocompatibility complex (MHC) upregulation. However, a reporter luciferase gene assay showed decreased activity for desialylated and biantennary sialylated IFN-β compared to the recombinant CHO-derived protein (Fig. 2B.1). This effect was not attributed to differences in thermostability or aggregation (Mastrangeli et al., 2015). Chemically synthesized IFN-β with a biantennary glycan, either sialylated or non-sialylated, demonstrated enhanced anti-tumor activity in a tumor mouse model. Notably, the sialylated synthetic IFN-β outperformed both asialo-IFN-β and commercially available CHO-derived IFN-β. Moreover, sialylation extended the plasma half-life of IFN-β after intravenous administration (Sakamoto et al., 2012). As glycosylation seems beneficial for IFN-β, further studies reported the introduction of a second *N-*glycosylation site at position Asn25 through an Arg27Thr mutation (new *N*-glycan site motif *N*-(X ≠ P)S/T) (Samoudi et al., 2017; Song et al., 2014). The resulting hyperglycosylated IFN-β demonstrated enhanced stability, extended plasma half-life, and reduced aggregation. However, despite maintaining receptor binding ability, it did not show any increase in bioactivity (Song et al., 2020; Song et al., 2014).

Lastly, similarly to GM-CSF, treatment with IFN-β induced an immune response, which was more pronounced for intravenously administered non-glycosylated variants (Durelli and Ricci, 2004; Fernández et al., 2001), but not after intramuscular injection (Deisenhammer, 2009); (Brickelmaier et al., 1999). Furthermore, non-native IFN-β could potentially trigger a cross-reactive response against native IFN-β (Brickelmaier et al., 1999). Together, these studies emphasize the importance of considering glycosylation when designing cytokine derivatives.

Overall, glycosylation on IFN-β enhances its solubility, stability, and biological activity. Furthermore, glycan structures, particularly sialylation and antennarity, play a significant role in improving or directing specific biological functions and activities (Fig. 4). Variants produced in *E. coli* showed reduced activity, increased aggregation and immunogenicity, while specific glycan modifications improve therapeutic efficacy and pharmacokinetics, highlighting the importance of glycosylation for IFN-β functionality.

### 1.4. Interferon-γ

Interferon-γ (IFN), also known as type II interferon, is a cytokine that aids in the defense against intracellular pathogens by activating macrophages, B cells, and DC. IFN-γ interacts with its receptor as a noncovalent homodimer (Lunn et al., 1992). It is involved in tumor immunology by stimulating the release of other pro-inflammatory cytokines and triggering immune responses against transformed cells. Dysregulation of IFN-γ results in autoimmune diseases. Although primarily produced by NK and natural killer T (NKT) cells, IFN-γ is also released by CD8 and CD4 Th1 effector T cells (Schoenborn and Wilson, 2007). Given its biological role, IFN-γ is an attractive cytokine in the development of cancer therapeutics and four immunocytokines have been reported (Ebbinghaus et al., 2005; Dougan et al., 2018; Chen et al., 2013; Hemmerle and Neri, 2014a) (Fig. 1, Table 1).

IFN-γ contains *N*-glycan sites on position Asn25 and Asn97 (Devos et al., 1982) (Fig. 3A, Table 2). The glycosylation pattern was first characterized when IFN-γ was isolated from stimulated human Peripheral Blood Mononuclear Cells (PBMCs) (Rinderknecht et al., 1984). IFN-γ is produced in two MW sizes: sequential Concanavalin A (ConA) lectin binding chromatography, followed by tryptic digestion, showed that the lower MW species carries a glycan on position Asn25, while the higher MW species is glycosylated on both *N*-glycan sites (Rinderknecht et al., 1984). In addition, a small quantity of non-glycosylated IFN-γ was found when PBMCs were stimulated with 12-O-tetradecanoylphorbol-13-acetate and phytohaemagglutinin (Kelker et al., 1984). Further analysis revealed microheterogeneity in the glycan structure with endogenous IFN-γ carrying mainly sialylated bi-antennary glycans. The mono- *vs* disialylated species ratio was around 3:2, mostly α2,6 linked, and corefucosylated. Different cell types, such as human monocytic cell line-HBL-38, secrete IFN-γ with similar glycosylation patterns as those produced by PBMCs with a slightly lower amount of di-sialylated glycan forms and more α2,3 linked-sialic acid, suggesting cell-type specific glycosylation patterns (Yamamoto et al., 1989). Indeed, IFN-γ derived from human T cells has glycan structures that differ per site. On the glycosylation site Asn25, complex bi-, tri-, and tetra-antennary structures terminating with sialic acid were identified, all carrying a corefucose. In contrast, on position Asn97 only a fraction of the detected *N-*glycans were core-fucosylated, with an absence of tetra- and triantennary glycans but an increase in hybrid and oligomannose structures. Both glycan sites were highly sialylated, in line with previous evidence (Mørtz et al., 1996a; Mørtz et al., 1996b; Sareneva et al., 1996). While a similar site-specific core fucosylation was observed for CHO-derived IFN-γ, its glycan profile was characterized by complex biantennary *N-*glycans varying in α2,3-linked sialylation and lacking α2,6 sialic acid, a feature common to CHO cells (Harmon et al., 1996; Hooker and James, 1998; James et al., 1995; James et al., 1996). IFN-γ has garnered significant interest as a model protein for studying various expression platforms, exploring different glycosylation profiles and investigating IFN-γ bioactivity (Figs. 2A, 3B) (Razaghi et al., 2016).

Glycans can facilitate IFN-γ secretion; tunicamycin treatment (Fig. 2C) of T cells reduced IFNγ secretion by 4–5 fold (Sareneva et al., 1996). Among the two glycosylation sites, Asn25 was found to be more effective in promoting IFN-γ secretion, while Asn97 had only a minor effect (Sareneva et al., 1994; Sareneva et al., 1996). Another study showed that non-glycosylated murine IFN-γ derived from CHO cells accumulated in the pre-Golgi compartment and was susceptible to misfolding and aggregation. This phenomenon was 9 times higher when cells were cultured at 40 °C. To a lesser extent this phenomenon was also observed for Jurkat and NK92MI which naturally produce human IFN-γ upon stimulation. During heat exposure, calreticulin, a calcium-binding chaperone protein located in the endoplasmic reticulum (ER), interacted with IFN-γ *N*-glycans to mediate proper protein folding. This interaction suggests that calreticulin monitors Asn25 glycosylation, which is crucial for successful IFN-γ secretion (Vandenbroeck et al., 2006). Remarkably, when IFN-γ was secreted without glycosylation, it was still able to form dimers, suggesting that glycosylation is dispensable for dimerization (Sareneva et al., 1994; Sareneva et al., 1996). In addition, glycosylation also influences IFN-γ stability. Computational models utilizing α2,6/α2,3-sialylated bi-antennary glycans, where Asn25 was core-fucosylated and Asn97 was not, confirmed that glycosylation is dispensable for IFN-γ biological activity, as their presence does not considerably change the protein secondary structure responsible for receptor-binding affinity. However, glycans were shown to limit the IFN-γ C-terminus unrestricted motion into the solvent, thus favoring conformations that are closer to the protein globule, thereby stabilizing the protein, shielding the cytokine from proteases and prolonging its active state (Lilkova et al., 2019) This evidence was supported by another study where glycans on Asn25, conferred protein protection against protease cleavage (*e.g.*, granulocyte protease, elastase, cathepsin G and plasmin) when compared to non-glycosylated *E.coli*-derived IFN-γ (Sareneva et al., 1995).

Overall, the presence of glycans on Asn25 can increase secretion and IFN-γ glycosylation seems to impact protein half-life by inhibiting proteolytic decay (Fig. 4). Based on the current understanding of the role of glycosylation in IFN-γ, it is important to consider the glycosylation status when developing treatment strategies involving this cytokine.

### 1.5. Tumor necrosis factor α

Tumor Necrosis Factor α (TNF-α) is a pleiotropic cytokine that is involved in several biological processes, spanning from inflammation and apoptosis to cell survival. TNF-α triggers signals *via* different pathways, including the NFkB pathway, MAPK pathway, and caspase-dependent apoptotic pathways each resulting in distinct cellular responses. In particular, TNF-α is a promising cytokine for tumor killing and has been utilized in the development of ten immunocytokines (Fig. 1, Table 1) (De Luca and Neri, 2018). While primarily produced by macrophages, TNF-α can also be expressed by other immune cell types and tissues (Ruddle, 2014; Sethi and Hotamisligil, 2021). Unlike many other cytokines, TNF-α is first produced as a type II transmembrane protein containing a 76 residues-long leader peptide at the N-terminus which includes a transmembrane domain (Table 2). Upon cell-surface expression, its extracellular domain forms a trimeric structure, subsequently cleaved off by disintegrin and metalloproteinase domain-containing protein 17 (ADAM17, also known as TNF-α converting enzyme, TACE) between Val(+1) and Ala(−1), facilitating its release from the membrane (Old, 1985).

The cDNA sequence did not reveal any potential *N*-glycosylation sites (Gray et al., 1990). It was later observed that 20 % of TNF-α derived from a human B cell line (BALL-1) was sensitive to neuraminidase treatment (Fig. 2B.2 and 0.4), suggesting that a portion of TNF-α was potentially modified with *O*-linked glycans containing sialic acid (Takakura-Yamamoto et al., 1996). A detailed peptide digestion analysis confirmed that Ser4 of the mature trimeric protein (157 aa, after cleavage by ADAM17) was occupied by an *O*-glycan of the mucin type carrying one sialic acid residue either α2,6 or α2,3-linked (Fig. 3, Table 2). Notably, the presence of α2,6 linkage to GalNAc was more prevalent than the α2,3 linkage to galactose (Takakura-Yamamoto et al., 1996). Almost 20 years later, it was reported that Human Embryonic Kidney (HEK) cells glycosylated TNF-α in a similar way (Wu and Robinson, 2022). An early study on TNF-α glycosylation estimated that only 49 % of the TNF-α trimers derived from a B cell line contained at least one *O*-glycosylated monomer (Takakura-Yamamoto et al., 1996). However, each HEK-derived recombinant TNF-α trimer carried at least one *O*-glycan. The authors also confirmed that the *O*-glycan was a disialyl-T antigen (Fig. 2B.4) (Wu and Robinson, 2022). The differences in the extent of *O*-glycosylation between these two studies may reflect the intrinsic disparities in *O*-glycosylation machinery and effector proteins regulating the process in the two different cell types.

Moreover, in BALL-1-derived TNF-α it was observed that *O*-glycosylation predominantly occurs on the mature form of TNF-α when it is cleaved at the canonical cleavage site between Val(+1) and Ala(−1) residues and to a lesser extent on the shorter variant which is cleaved after Ser3. The authors hypothesized that the amino-terminal domain could be a poor substrate for *O*-GalNAc transferases, explaining the incomplete glycosylation (Takakura-Yamamoto et al., 1996). Later it was observed that the presence or absence of *O*-glycosylation on Ser80 (which corresponds to Ser4 in the mature TNF-α) affects its cleavage by ADAM17 and other proteases of the ADAM family. Specifically, when Ser80 is glycosylated with one GalNAc *O*-glycoform by GalNAc-T2, it is partially protected from ADAM17 cleavage, leading to an increase of free TNF-α *in vitro* in GalNAc-T2 knock-out (KO) HepG2 and HEK cell lines, as well as *in vivo* Galnt2−/−KO mice (Goth et al., 2015). It is noteworthy that O-glycosylation on the same Ser80 residue completely avoids the cleavage by ADAM12, preventing the generation of the shorter form of TNF-α. This is in agreement with earlier findings (Takakura-Yamamoto et al., 1996) where non-glycosylated species contained a truncated N-termini starting with Ser, suggesting that also ADAM12 is involved in TNF-α release to a certain degree (Goth et al., 2015; Takakura-Yamamoto et al., 1996). Lastly, a native top-down mass-spectrometry study by Wu et al. implied that the *O*-glycan is required to stabilize TNF-α as a dimer, thereby contributing to forming the final stable trimer, supporting the role of the *O*-glycan in facilitating oligomerization (Takakura-Yamamoto et al., 1996).

According to the current knowledge, on one side interfacial *O*-glycosylation on TNF-α seems to stabilize the homotrimeric architecture, on the other, it has a negative co-regulative effect as it seems to decrease, the amount and speed of TNF-α shedding from the membrane, when both GalNAcT2 and specific ADAM proteases are co-expressed (Fig. 4). Hence, while the presence of the glycans may be critical in prolonging TNF-α activity and long-term storage in the context of using TNF-α as a recombinant therapeutic protein, its overall production can be limited in yield based on the specific chosen recombinant host (Goth et al., 2015; Takakura-Yamamoto et al., 1996). It is important to underline that with the advent of new fusion proteins and immunocytokines, *O*-glycosylation on TNF-α can be a tool for stabilizing and maintaining fusion between different protein partners.

### 1.6. Interleukin-2

Interleukin-2 (IL-2), also known as T-cell growth factor (TCGF), is mainly secreted by T cells upon stimulation and augments cytotoxic T-cells, NK cell activity, proliferation, and differentiation of B-lymphocytes. In low doses, IL-2 can also stimulate regulatory T-cell populations. Its pro-inflammatory role provides therapeutic activity against cancer and led to FDA approval for treating melanoma and renal cell carcinoma in 1992 and 1998, but soon later withdrawn for its severe side effects (Hernandez et al., 2022). However, IL-2 has recently gained interest (Mullard, 2021) and many engineering strategies focused on redesigning IL-2, resulting in new IL2 Fc-fusion or albumin-fusion proteins, IL-2 immunocytokines, pegylated IL-2, IL2 muteins and neoleukins (Fig. 1, Table 1) (Codarri Deak et al., 2022; Holder et al., 2022; Silva et al., 2019). Therefore a better comprehension of IL-2 biology, especially its glycosylation could prove instrumental in refining these new approaches.

IL-2 was initially reported to not contain glycans, as there was no retention after lectin-coupled affinity column purification (Mier and Gallo, 1980). Later, scientists showed that different cell lines release a mixture of different MWs IL-2 upon diverse stimuli. PHA-stimulated lymphocytes produce IL-2 in two molecular sizes of 16 and 17 kDa, while Burkitt’s lymphoma cell line Daudi, produces IL-2 of 14 kDa (Mier and Gallo, 1980; Welte et al., 1982). This apparent heterogeneity was later explained by the presence of glycosylation and sialylation patterns on IL-2 derived from normal human tonsil and Jurkat cells. Neuraminidase treatment of tonsil-derived IL-2 confirmed the presence of sialic acid (Fig. 2B.1 and 0.4) (Robb and Smith, 1981). While the cDNA of human IL-2 from Jurkat cells confirmed the absence of potential *N-*glycosylation sites (Robb et al., 1984; Taniguchi et al., 1983), researchers later identified that IL-2 contains one *O*-glycan on Thr3 (Fig. 3, Table 2) (Robb et al., 1984; Taniguchi et al., 1983). Human lymphocytes from healthy donors produced IL-2 with mono or di-sialylated *O-*glycans, whereas Jurkat-derived IL-2 carried predominantly non-sialylated *O*-glycans (Fig. 2B.4) (Conradt et al., 1985). To elucidate IL-2 glycosylation in mammalian cell expression platforms, different systems were chosen to express IL-2 (Fig. 3B). Interestingly, *O-*glycosylation on IL-2 when expressed in BHK, Ltk-, and CHO cells, revealed an identical type of *O-*glycan on Thr3 as observed for T-cell derived IL-2. Each recombinant mammalian cell line secretes glycosylated neutrally charged, mono- or di-sialylated, and non-glycosylated IL-2 at a constant ratio in both high and low-producer clones. However, while Ltk- and BHK cells secrete 40–60 % of the total IL-2 in glycosylated form, CHO cells predominantly secrete the glycosylated IL-2 form (> 90 %) (Conradt et al., 1989).

To explore the biological effect of IL2 glycoforms, the three different glycosylated CHO-derived IL-2 species were isolated using cation-exchange high performance liquid chromatography and tested for activity on a murine IL-2-dependent cytotoxic T cell line. They all showed similar activities, suggesting that their interaction with the IL-2 receptor to induce proliferation *in vitro* might be comparable (Marchese et al., 1990). However, observations were reported indicating that non-glycosylated *E-coli*-derived IL-2 exhibited a decreased ability to support clonal outgrowth and long-term propagation *in vitro* of alloactivated human T-lymphocytes, suggesting that glycosylated natural PBMC-derived IL-2 demonstrates certain activity and may be preferable for cloning and long-term cultivation of T cells (Pawelec et al., 1987; Roifman et al., 1985).

The initial studies on IL-2 as a pioneer in immunotherapy were hampered as severe side effects emerged. New engineering strategies are currently exploring alternatives to address the limitations (Hernandez et al., 2022; Mullard, 2021). For example, an immunocytokine fusion protein was developed, comprising an anti-carcinoembryonic antigen (CEA)-specific antibody fused with an IL-2 variant. In this construct, the *O-*glycosylation site was eliminated by replacing Thr with Ala (Thr3Ala) to ensure uniformity (Klein et al., 2017). Similarly, clinically used IL-2 is mainly produced in *E. coli* and does not have *O-*glycosylation but further work would be beneficial to test if *O-*glycans could be used to develop and refine novel therapeutic formats.

### 1.7. Interleukin-3

IL-3 is a multifunctional cytokine (multi-CSF) produced by activated T cells, monocytes, NK cells, mast cells, keratinocytes, and endothelial cells. It binds to a low-affinity IL-3 α receptor (CD123) and a shared β receptor with GM-CSF and IL-5 (Gianella-Borradori, 1994; Mangi and Newland, 1999). IL-3 stimulates multipotent erythroid and myeloid progenitor cells to proliferate and differentiate into various blood and immune cells (Eder et al., 1997). IL-3 is not primarily involved in steady-state hematopoiesis but acts as an early-acting factor at inflammation sites, playing a role in autoimmune diseases, cancer, and infections (Dougan et al., 2019; Podolska et al., 2024). IL-3 exhibits similar activity to GM-CSF and G-CSF, aiding in the recovery of neutrophils to treat cytopenia. However, recombinant IL-3 alone did not significantly reduce cytopenia in chemotherapy-treated patients compared to GM-CSF and G-CSF (Palmeri et al., 1999). Therefore, fusion proteins involving IL-3 and GM-CSF have been designed to prevent thrombocytopenia in chemotherapy-treated patients with certain cancer types (Curtis et al., 1991; Vadhan-Raj et al., 1994; O’Shaughnessy et al., 1996). Additionally, IL-3 has been combined with a truncated diphtheria toxin domain as a payload to specifically target CD123-expressing leukemic stem cells (Fig. 1, Table 1) (Syed, 2019). Given its pivotal role in hematopoiesis, the production of recombinant IL-3 is expanding for research applications and new engineering strategies.

The human IL-3 cDNA revealed two putative *N*-glycosylation sites at residues Asn15 and Asn70 (Fig. 3, Table 2). In monkey COS-1 cells, IL-3 was secreted as a mixture of different MWs ranging from 15 to 25 kDa, demonstrating varying degrees of *N*-glycosylation (Yang et al., 1986). Similarly, insect-derived IL-3 exhibited MW ranging from 15 to 21.5 kDa. The first glycosylation site of insect-derived IL-3 was fully glycosylated, while the second site was glycosylated only about 35 % as determined by mass spectrometry. Specifically, paucimannosidic structures were identified at both glycosylation sites, with the majority showing proximal bifucosylated trimannosyl structures (60 %), and fewer (40 %) lacking one fucose or one mannose residue, typical for recombinant proteins produced in insect cells (Fig. 2A and B.2)(Ding et al., 2003). *P. pastoris*-derived IL-3 was primarily produced in molecular species of 19 and 22 kDa, slightly different from recombinant insect-derived IL-3 (15–25 kDa), suggesting differences in glycosylation between the two expression systems (Fig. 2A). PNGase F treatment of the yeast-derived IL-3 revealed that the two sites are occupied to varying degrees (Li et al., 2011), but no further glycan characterization was carried out.

As indicated before IL-3 has been expressed in various systems, including mammalian (Yang et al., 1986), bacteria (Dagar and Adivitiya, 2017; Davis et al., 1999; Hercus et al., 2013; Nguyen et al., 2024), yeast (Dagar et al., 2016; Dagar et al., 2022; Li et al., 2011), and insect cells (Fig. 3B) (Ding et al., 2003). Non-glycosylated IL-3 expressed in *E. coli* often resulted in inclusion bodies due to protein aggregation and several strategies have been attempted to increase its solubility using fusion tags (Dagar and Adivitiya, 2017; Davis et al., 1999; Hercus et al., 2013) (Nguyen et al., 2024). Insect cells-produced IL-3 showed higher specific biological activity by [^3^H]thymidine incorporation and WST-1-assay compared to *E. coli*-derived IL-3. The authors speculated that the non-glycosylated commercial product was overpacked to compensate for lower specific activity as it may accumulate in an insoluble form (Ding et al., 2003). The role of glycosylation in *P. pastoris-*derived IL-3 was further elucidated by site-directed mutagenesis, revealing its impact on cytokine activity, stability, and secretion. The yeast-derived mutant proteins (Asn15Ala, Asn70Ala, Asn15/70Ala) showed slightly better proliferation using MTT assay on TF-1 cells compared to fully glycosylated yeast-derived recombinant IL-3. However, all IL-3 mutants significantly decreased protein secretion in *P. pastoris* compared to the fully glycosylated control. Furthermore, glycosylated IL-3 showed higher thermal stability than its non-glycosylated counterpart (N15/70A) but similar thermal stability to variants with a single glycosylation site (N15A, N70A)(Fig. 4)(Dagar et al., 2022).

IL-3 represents an interesting model cytokine where glycosylation can affect protein solubility, secretion, and thermal stability while minimally impacting cytokine-specific activity as previously reported in the pioneering studies performed on recombinant murine IL-3 (Ziltener, 1993). Further glycan characterization of the native IL-3, together with complementary *in vivo* studies would help to elucidate its role in protein half-life and immunogenicity.

### 1.8. Interleukin-4

Interleukin-4 (IL-4) is an essential cytokine in the fight against parasite infections, but it is often dysregulated in allergic diseases like asthma. Primarily produced by mast cells, eosinophils and basophils, IL-4 promotes the proliferation of Th2 cells and B cells (Steinke and Borish, 2001). A fusokine of IL-4 with IL-10 has been developed to suppress inflammatory cytokines and reduce chronic pain (Fig. 1, Table 1) (Eijkelkamp et al., 2016; Steen-Louws et al., 2019a). This new cytokine fusion format has been extensively investigated to assess the impact of glycosylation on its therapeutic potential.

IL-4 contains two potential glycan sites Asn38 and Asn105 (Fig. 3A, Table 2). When IL-4 is expressed in *Saccharomyces cerevisiae (S. cerevisiae)* it is hyperglycosylated, primarily with oligomannose, leading to a 3–6 times increase in MW compared to the non-glycosylated IL-4 (Fig. 2A) (Solari et al., 1989). However, when expressed in a different yeast strain, *P. pastoris,* or Cl27 mouse mammary tumor cells, IL-4 is glycosylated only on Asn38 (Carr et al., 1991). Glycan profiling of HEK293-derived IL-4 revealed complex bi-antennary glycans with fucose and sialic acid (70.39 %) (Lee et al., 2021). Unfortunately, no additional literature is available on endogenous IL-4 glycosylation.

Despite the lack of comprehensive IL-4 glycan characterization, glycosylation on *S. cerevisiae-*derived IL-4 does not affect the cytokine biological activity as determined by receptor binding studies using Raji cells. Furthermore, hyperglycosylated and *N*-glycanase-treated *S. cerevisiae*-derived IL-4 were equally able to induce the proliferation of PHA-activated T-cells and the upregulation of CD23 on monocytes and human tonsillar B cells when co-stimulated with IgM (Solari et al., 1989). In contrast, the *P. pastoris-*produced IL-4 exhibited slightly lower biological activity compared to the PNGase F-treated IL-4 when tested for their ability to induce CD23 on human B cells. This difference may be attributed to *P. pastoris* production of core *N*-glycans with larger phospho-mannans compared to *S. cerevisiae*. This peculiar feature influenced the stability of IL-4 in culture medium under different temperature conditions and over an extended time: the glycosylated IL-4 from *P. pastoris* showed shorter half-life compared to the non-glycosylated IL-4, due to rapid degradation by cells in culture (Li et al., 2014).

The fusokine of IL-4 with IL-10 was expressed in HEK293 cells and CHO cells co-transfected with α2,3-sialyltransferase to generate two distinct glycoforms with different sialylation levels: IL-4–10FP lowly sialylated (lowSA) and highly sialylated (highSA) (Eijkelkamp et al., 2016; Steen-Louws et al., 2019b) Both variants demonstrated similar *in vitro* and *in vivo* potency by inhibiting LPS-induced TNF production in whole blood, and by suppressing inflammatory hyperalgesia after intrathecal administration in a mice carrageenan-induced inflammatory pain model. Although the different sialylation pattern did not affect the anti-inflammatory properties, the systemic exposure of the two glycoforms *in vivo* determined different clearance rates, with IL4–10 FP low SA cleared more rapidly, as it may interact with asialo-glycoprotein receptor (ASGPR) binding to galactoses.

Overall, the role and bioactivity of glycans on IL-4 remain unclear, but they may influence cytokine half-life (Fig. 4). Further research is needed especially to characterize the endogenous glycan forms produced by human immune cells. While glycosylation seems dispensable for cytokine bioactivity *in vitro* as shown when testing IL-4 derived from different expression platforms, it is still unknown if the *N*-linked glycans produced in human or mammalian hosts are required for IL-4 expression, secretion, and activity *in vivo.*

### 1.9. Interleukin-6

Interleukin-6 (IL-6) is a cytokine with various functions depending on the target cell type. IL-6 can stimulate B cells leading to antibody production or induce T cell activation (Kang et al., 2020). IL-6 is currently investigated in preclinical studies as a fusokine with ciliary neurotrophic factor (CNF) for the potential treatment of type 2 diabetes (Fig. 1, Table 1) (Findeisen et al., 2019).

IL-6 can be both *N*- and *O-*glycosylated with two potential *N*-glycan sites at positions Asn46 and Asn145 (Hirano et al., 1986) and one *O*-glycosylation site on Thr139 (Orita et al., 1994) (Fig. 3A, Table 2). The extent and type of glycosylation vary based on the cell source. In B cell lines (sfBJAB, RPMI1788, TM-1, and HS-2), IL-6 is primarily non-glycosylated, while IL-6 released from IL-1β-stimulated monocytes, PBMCs and fibroblast cells exhibits varying degree of glycosylation, resulting in molecules with different MW (May et al., 1991b) (Fig. 3B). The higher MW forms of IL-6 are *N-*glycosylated, as monocytes treated with tunicamycin were unable to produce them (Fig. 2C) (May et al., 1991a; Tanner et al., 1990). While the lower MW forms of IL-6 isolated from PBMCs and fibroblasts were confirmed to carry *O*-glycosylation after endoglycosidases treatment (Gross et al., 1989; May et al., 1989; May et al., 1991a; May et al., 1991b; Santhanam et al., 1989; Tanner et al., 1990). It should be noted that IL-6 can have two distinct N-termini from differences in signal peptide cleavage in humans. When starting with Ala IL-6 undergoes only *O-*glycosylation, while when starting with Val, both *N-* and *O*-glycosylation occur (Hasegawa et al., 1992; May et al., 1991b).

Upon close investigation of the glycosylation types found on CHO-derived IL-6, *O-*glycans were identified as di-sialylated core1 *O*-glycan (Fig. 2B.4) (Orita et al., 1994), while *N*-glycans were only found at position Asn46 rather than Asn145 and exhibited a complex *N-*glycan type (Hasegawa et al., 1992; Orita et al., 1994). *N*-glycan profiling revealed the presence of fucosylated bi- or tetra-antennary *N-*glycans, eventually with one or two *N-*acetyllactosaminyl repeats (Fig. 2B.1 and 0.2) (Orita et al., 1994). In contrast, *N*-glycan profiling of IL-6 derived from human primary monocytes revealed the presence of di- and less abundant triantennary *N*-glycans capped with various levels of sialic acid (53 % sialylated and ≈6 % neutral) or oligomannose-type structures (40 %) (Parekh et al., 1992). Among the mannosidic *N*-glycans, paucimannose (Man2) was predominant (32 %), followed by the high mannose glycans including Man5 or Man6 (4 % each) and traces of Man8 (Fig. 2B.2). In addition, a small population of core-fucosylated or mono-galactosylated bi-antennary *N*-glycans was identified (Parekh et al., 1992; Reif et al., 2021).

Mammalian cell-derived IL-6 (COS-7, PA317 and GH3 cells) showed a greater ability to promote the growth and survival of IL-6 dependent B9 hybridoma cells *in vitro* than *E. coli*-derived IL-6. The difference in bioactivity was at least one order of magnitude depending on the mammalian cells expressing IL-6 (COS-7 2 × 10^9^ U/mg, PA317 1.75×10^8^U/mg, GH3 2.3 × 10^8^ U/mg *vs* 4.4×10^4^U/mg E coli derived IL-6) (Tanner et al., 1990). Strikingly, non-glycosylated *E. coli-*derived IL-6 had reduced capacity to induce a rise in body temperature in a pyrogenic *in vivo* rabbit model compared to glycosylated human fibroblast-derived IL-6 (May et al., 1989). These findings indicate that the glycans may influence IL-6 activity, however, other modifications that are missing in *E. coli*-derived IL-6, such as phosphorylation and oligomerization, may additionally impact its biological activity (May et al., 1991a; May et al., 1989).

To investigate the bioactivity, nine different IL-6 isoforms representing the most and minor abundant *N*-glycans found on the cytokine were semi-synthetized by chemically coupling glycans to Asn46. All of them were equally active in sustaining the proliferation of IL-6-dependent Ba/F3-gp130-hIL-6R cell lines. Surprisingly, the activity of the individual glycoforms was comparable to the non-glycosylated *E. coli*-derived IL-6. While the single different *N-*glycan structures do not affect IL-6 binding, the authors observed significant differences in the plasma half-life in rats for 7 of the glycoforms and *E. coli*-derived IL-6, suggesting that glycosylation could impact therapeutic efficacy *in vivo*. The shortest half-life was reported for the *E. coli*-derived IL-6, paucimannose (Man2) and the bi-antennary α2,6-sialylated glycoforms while IL-6 with Man5-glycan and α2,3-sialylated bi-antennary glycans showed longer half-life. Bi-modal half-lives were reported for the IL-6 tetra-antennary complexes, a glycoform designed to hypothetically maximize cytokine half-life in combination with sialylation/desialylation. Notably, the galactose-capped tetra-antennary complex glycoform had the slowest clearance rate, which is usually associated with the sialylated glycoform instead (Reif et al., 2021).

To summarize, while IL-6 *N*-glycans have been extensively investigated, there is a lack of understanding of the functional role of *O*-glycosylation on IL-6 (Gross et al., 1989; Santhanam et al., 1989; Tanner et al., 1990). *N-*glycans affect the half-life of IL-6 *in vivo,* with larger α2,3-sialylated branched *N-*glycans as the most effective strategies in prolonging cytokine half-life (Fig. 4). Additionally, other modifications, such as oligomerization and phosphorylation, can have an impact on IL-6, making the choice of the production platform decisive. Further understanding of the role of glycosylation on IL-6 and its heterogeneity is required to provide valuable insights on how to improve its therapeutic potential. By properly choosing the production platform and engineering glycans on IL-6, it is possible to foresee the development of more effective IL-6-based therapeutics.

### 1.10. Interleukin-7

Interleukin-7 (IL-7) has multiple roles in T cell biology, including the survival, proliferation, and differentiation of developing thymocytes in the thymus, as well as survival and homeostasis of naïve T cells and memory CD8+ T cells in the periphery. Additionally, IL-7 is a key player in the differentiation of immature pre−/pro-B cells (Corfe and Paige, 2012). IL-7 has emerged as a promising target to treat immunodeficiencies such as those resulting from chemotherapy, stem cell transplantation, and HIV infection (Mackall et al., 2011). Moreover, like other cytokines, new engineering approaches are currently tested in cancer immunotherapy, including IL-7 combination with the Fc domain of different antibodies (*e.g.*, anti-PDL1) (WO2019144945A1), antibody fragments (Pasche et al., 2011) or other cytokines (*e.g.*, GM-CSF, IL-15) (Fig. 1) (Hsieh et al., 2015; Song et al., 2016).

IL-7, initially known as lymphopoietin-1 (LP-1), was initially produced in COS-7 cells (Goodwin et al., 1989; Namen et al., 1988), where the cDNA revealed three potential *N*-linked glycosylation sites at Asn70, Asn91 and Asn116 (Goodwin et al., 1989). In insect cells, IL-7 was expressed as a single band of 24 kDa (Mirzaei et al., 2008), while in CHO-K1 cell lines as a single band of 25 kDa, suggesting that IL-7 undergoes glycosylation as the non-glycosylated protein has a MW of 17 kDa (Toghraie et al., 2017). However, none of these studies provide a thorough characterization of IL-7 glycosylation or bioactivity. The only glycoprofile was reported for CHO-derived IL-7 which shows a mixture of bi- and triantennary *N*-glycan structures capped with varying levels of sialic acid. In addition, a lectin binding analysis indicates the presence of sialylated *O*-glycan structures, presumably on Thr111 (US7708985B2). Characterization of the yeast-derived IL-7 showed that the cytokine is hyperglycosylated (~40–80 kDa) on the predicted *N-*glycan sites as PNGase F treatment led to a consistent shift in MW (~17 kDa).

Several expression systems have been tested to produce IL-7, including *E. coli* (Ouellette et al., 2003), yeast (Luo et al., 2009), insect (Mirzaei et al., 2008) and mammalian cells (Toghraie et al., 2017) (Fig. 3B, Table 2). *E. coli-*derived IL-7 is not glycosylated and shows comparable biological activity to IL-7 generated from *P. pastoris* in an IL-7 dependent murine pre-B cell line (2E8) proliferation assay (Ouellette et al., 2003). However, in a distinct follow-up study, the authors underlined the importance of performing extensive protein deglycosylation of *P. pastoris*-derived IL-7 prior to *in vitro* activity assessment (Fig. 2C) (Zaremba-Czogalla et al., 2015).

*N*- and *O*-linked glycosylation in proteins may confer increased resistance to proteolysis (Goth et al., 2018; Rudd et al., 1994). Neutrophil enzymes, specifically matrix metalloprotease-MMP-9, cleave human IL-7 within a protease-sensitive loop, located between helix C and D. Glycosylated insect and HEK293 cell-derived IL-7 showed reduced proteolytic degradation compared to the non-glycosylated *E. coli*-derived IL-7, suggesting that *N-*glycosylation may play a protective role on IL-7. However, a 4-fold decreased binding to IL-7 receptor alpha was observed for the glycosylated IL-7 compared to *E. coli* derived IL-7 (Vandooren et al., 2021).

Overall, IL-7 production has been pursued in various host systems: while non-glycosylated IL-7 retains biological activity, glycosylated IL-7 may offer an advantage by enhancing its resistance to proteases (Fig. 4). Further exploration of the *N*- and the less known *O*-glycosylation can help additionally fine-tuning IL-7 properties to advance new therapeutics development.

### 1.11. Interleukin-9

Interleukin-9 (IL-9) shows pleiotropic activity (Uyttenhove et al., 1988) and is important in regulating inflammatory immunity. IL-9 is primarily produced by T-lymphocytes, but evidence suggests that other T helper cell subsets, including the Th17 phenotype, can produce it. T regulatory cells also express IL-9 suggesting that this cytokine can be both pro-inflammatory and anti-inflammatory (Goswami and Kaplan, 2011). IL-9 interacts with different cell types like B cells, mast cells, and innate lymphoid cells (ILCs) (Goswami and Kaplan, 2011). IL-9 has also been described in an immunocytokine format targeting the fibronectin EDA which is used in pulmonary hypertension and expressed in CHO cells (Fig. 1, Table 1) (Heiss et al., 2022).

In 1990, the human IL9 cDNA (p40) was isolated (Renauld et al., 1990) and four putative *N*-glycan sites were identified at Asn32, Asn45, Asn60 and Asn96 (Fig. 3A, Table 2). When expressed in Sf9 insect cells, IL-9 resulted in a 23 kDa protein, higher in MW compared to the predicted 14 kDa non-glycosylated protein, indicating that the cytokine is highly glycosylated (Renauld et al., 1990). However, limited information is currently available on the glycosylation of IL-9, as it has not been glycoprofiled and the functional effect of the glycans has not been investigated. Therefore, it remains unclear whether glycans on IL-9 have an impact on its folding, stability or biological activity, and further understanding may be critical given the growing interest in IL-9 for therapeutic strategies (Fig. 4).

### 1.12. Interleukin-12

Interleukin-12 (IL-12) is a heterodimeric cytokine consisting of two subunits, p40 and p35, and belongs to a cytokine-family with similar structures including IL-23, IL-27, and IL-35. IL-12 induces IFN-γ production, proliferation of activated T and NK cells, and Th1 differentiation. IL-12 has shown potent antitumor and antimetastatic activity. Even though its toxicity has limited its clinical use, various strategies are being developed to reduce its toxicity while maintaining its bioactivity (Pires et al., 2021). Numerous therapeutic formats have been designed, such as 9 distinct immunocytokine formulations and a supercytokine variant of IL-12 (Fig. 1, Table 1) (Greiner et al., 2021; Liu et al., 2017; Morillon 2nd et al., 2019).

IL-12 cDNA, encoding for the two separate subunits, was isolated from EBV-transformed B cell line (RPMI 8866) revealing the presence of multiple *N-*glycosylation sites (Wolf et al., 1991). IL-12α has two potential *N*-glycan sites, one at position Asn71 and one at Asn85. Their mutation to Gln in HEK293T cells resulted in a protein with MW similar to PNGase F treated IL-12α, thus confirming the *N*-glycan sites occupancy. IL-12β has 4 potential *N-*glycans sites at positions Asn103, Asn113, Asn200 and Asn281 (Fig. 3A, Table 2). Although Asn103 and Asn200 were predicted to be occupied (NetNGlyc-1.0) (Gupta and Brunak, 2002), only Asn200 was experimentally validated by site-directed mutagenesis. Analysis of the single mutant IL-12β Asn200Gln revealed that Asn113 and Asn281 can also be occupied to a certain extent (Bohnacker et al., 2020). Interestingly, IL-12β contains additional sites for tryptophan *C*-mannose glycosylation on Trp297/300 (Doucey et al., 1999). Furthermore, COS7-derived IL-12α subunit is highly sialylated as shown after neuraminidase treatment. Upon exposure to different glycosidases, distinct glycosylation phenotypes were observed in the two subunits (Fig. 2B.2). IL-12α did not show susceptibility to endoglycosidase (Endo) H or *O-*glycosidase treatment after secretion (Fig. 2B.2 and 0.4), suggesting that IL-12α is characterized by complex-type glycans, whereas IL-12β was sensitive only to Endo H and not to Endo F, suggesting the presence of high mannose sugars (Murphy et al., 2000). Further mass-spectrometry validation is needed to completely characterize the glycosylation pattern on both IL-12 subunits.

The role of glycosylation on IL-12 and the related cytokine family, including IL-23, IL-27 and IL-35, was studied on glycoforms obtained by site-directed mutagenesis. It is noteworthy that the secretion of IL-12 hinges on the simultaneous assembly of both its α and β subunits (Bohnacker et al., 2020). In COS7-derived IL-12α glycosylation was found to be a prerequisite for secretion (Murphy et al., 2000). However, the loss of glycosylation does not seem to severely affect HEK293T-derived IL-12 and IL-23 secretion or heterodimer formation, in contrast to IL-35 where non-glycosylated IL-12α and EIB3 are retained in the ER (Bohnacker et al., 2020). Furthermore, *in vitro* CD14-depleted PMBCs cell proliferation and in iLite(R) receptor assays, the presence of glycans on IL-12 subunits did not significantly affect its biological activity (Bohnacker et al., 2020).

Unraveling the impact of *C*-mannosylation on IL-12β showed that the majority of *P. pastoris*-derived IL-12β exists in the doubly *C-*mannose glycosylated form, supporting increased resistance to thermal denaturation compared to its non-glycosylated counterpart (~ΔT_m_ = 5 K). Notably, the expression of IL-12β was 2 times higher in *P. pastoris* that stably expressed *Caenorhabditis elegans* DPY-19, a *C-*mannosyltransferase, when compared to the wild-type strain (John et al., 2021).

To evaluate the protein glycosylation effect for the development of antibody-based biotherapeutics, Bootz et al. investigated the suboptimal tumor-targeting properties of a CHO-expressed fusion construct containing the IL-12β murine subunit (p40) at the C-terminus of a F8 diabody. Despite the murine origin of IL-12β, the 3 *N*-glycan sites are conserved within the human IL-12β (Asn103,113 and 200) amino acid sequence. Interestingly, varying glycan structures on the murine p40 resulted in different tissue distributions. While the removal of up to 3 *N*-glycosylation sites did not lead to the recovery of disease *in vivo*, almost complete recovery was achieved with enzymatically PNGase F deglycosylated protein preparation (Bootz et al., 2016).

The complexity of IL-12 subunit assembly-induced heterodimerization generally requires a eukaryotic expression platform where differences in glycosylation pattern can have an impact on IL-12 fusion construct stability and bioavailability (Fig. 4).

### 1.13. Interleukin-13

Interleukin-13 (IL-13) is primarily secreted by activated Th2 subset cells (Minty et al., 1993) and plays its immunomodulatory role by inhibiting the production of pro-inflammatory cytokines (*e.g.*, IL-1, IL-6, IL-8, IL-12) on different cell types, including B cells, monocytes, macrophages, and endothelial cells (Punnonen et al., 1993). IL-13 is also responsible for antibody isotype class switching to IgE along with IL-4 and for stimulating B lymphocytes. These combined features make IL-13 an attractive candidate for treating different pathological conditions from cancer to chronic inflammatory diseases (Wang et al., 2008)**.** In particular, IL-13 has been explored in the treatment of cancer in the formulation of supercytokines (Fig. 1, Table 1)(Zheng et al., 2022).

The cDNA isolated from human PBMCs revealed the presence of four *N-*glycan sites Asn28, Asn39, Asn47, and Asn62, which were confirmed to be occupied after transfection of COS-7 cells with tunicamycin (Figs. 2C, 3A) (Minty et al., 1993). However, IL-13 secreted by human T cells is around ~10 kDa indicating little to no *N*-linked carbohydrate (McKenzie et al., 1993), a characteristic also observed in the NS-0 rodent cell line (Eisenmesser et al., 2000). Tobacco plant-derived human IL-13 exhibited multiple molecular forms due to different *N*-linked glycosylation patterns, sensitive to both PNGase F and Endo H treatment (Fig. 2B.2). Notably, Endo H digestion was only partial, indicating that IL-13 carries predominantly complex type *N-*glycans (Wang et al., 2008) but no further glycan characterization on IL-13 was carried out. *N-*glycan analysis of HEK-derived IL-13 showed that complex biantennary glycans predominate with Asn37 being the most *N*-glycosylated and Asn52 containing the highest abundance of tri- and tetra-antennary *N*-glycans. Only 48 % of *N*-glycopeptides contain a core-fucose and sialic acid. *N-*glycans do not affect bioactivity as *N-*glycosylated or tunicamycin treated (non-glycosylated) COS-7-derived IL-13 showed similar TF-1 cell proliferation ability (McKenzie et al., 1993). Similarly, expression of IL-13 in the NS-0 cell line resulted in biologically active non-glycosylated cytokine capable of inducing TF-1 cell proliferation (McKenzie et al., 1993), as for *E. coli-*derived IL-13, confirming that glycosylation is dispensable (Eisenmesser et al., 2000). Notably, plant-derived IL-13 exhibited greater resistance to trypsin, pepsin and pancreatin digestion compared to *E. coli-*derived IL-13 (Fig. 4), suggesting that glycans play a protective role, showcasing a potential for its oral delivery in the treatment of gastrointestinal nematode infection or type 1 diabetes (Wang et al., 2008).

Additional insights on IL-13 glycosylation and its correlation with protein bioactivity and protease degradation may help in designing new therapeutic antibody-fusion constructs.

### 1.14. Interleukin-15

Interleukin-15 (IL-15) is a proinflammatory cytokine that shares biological functions with IL-2 in the regulation of both innate and adaptive immune responses. Unlike IL-2, IL-15 is constitutively expressed in various tissues, and it does not promote the survival of CD4+ CD25+ regulatory T cells for immunosuppression, but it supports the survival of CD8+ memory T cells and stimulates NK cells (Tan and Lefrançois, 2006; Huang et al., 2018). IL-15 serves as a broad proinflammatory modulator and it holds promise for applications in cancer immunotherapy (Fig. 1, Table 1).

The cDNA indicated the presence of three putative *N-*glycosylation sites from the sequence without propeptide: Asn71, Asn79 and Asn112 (Fig. 3, Table 2) (Grabstein et al., 1994). Glycan analysis performed on IL-15 co-expressed with IL15Rα in HEK293 cells showed that IL-15 carries predominantly bi-antennary complex type glycans with core-fucose, with or without bisecting *N*-acetyl glucosamine (GlcNAc) and with low amounts of galactosylation and sialylation. Furthermore, the study revealed that Asn79 is occupied (for 95 %) whereas Asn71 and Ans112 are not (Thaysen-Andersen et al., 2016). In contrast, when IL-15 was fused to an ABD scFv at its C-terminus and expressed in HEK293, all its putative glycosylation sites were occupied (Huang et al., 2018; Tan and Lefrançois, 2006). In *P. pastoris*, IL-15 is glycosylated, albeit the increased size compared to the CHO-derived IL-15 (Xue et al., 1999) is most likely due to oligomannosidic structures (Fig. 2A). However, a thorough glycosylation characterization for yeast-derived IL-15 is missing (Sun et al., 2016).

Regarding the glycan function, molecular insights on IL-15 and IL-15 receptor interactions, both expressed in *E.coli*, showed that Asn71 located at the end of helix C, is adjacent to Asn72 (putative *N*-glycan site), whose mutation to Asp affects IL15-Rβ interaction (Chirifu et al., 2007; Huang et al., 2018), resulting in an IL-15 mutein with agonist activity (Chirifu et al., 2007; Huang et al., 2018). However, the most likely occupied glycan site Asn79, located at the end of the C-D loop, is far away from the receptor binding sites, while Asn112, located at the end of helix D, is predicted to form a hydrogen bond with Tyr103 of the IL-15γ chain receptor. The distance of Asn79 from the probable receptor binding sites is unlikely to affect receptor interaction strongly, but their presence might decrease their “on rate” for receptor binding (Chirifu et al., 2007; Huang et al., 2018).

In mammalian expression systems, the low IL-15 expression was overcome by co-expression of IL15Rα or by fusion with antibody, antibody fragments, or binding domains. Huang et al. successfully produced IL-15 fused with an ABD scFv at its C-terminus in HEK293 cells (Fig. 1, Table 1) (Chirifu et al., 2007; Huang et al., 2018). Following site-directed mutagenesis to remove all 3 *N*-glycosylation sites in IL-15, the resulting mutant was poorly expressed, suggesting that glycosylation may be necessary for efficient production in HEK293T cells. By testing the bioactivity, the presence of the glycans on IL-15 accounted for a loss of 75 % in the ability to stimulate CTLL2 T cell lines, compared to the same molecule after PNGase F deglycosylation. Moreover, the single mutants Asn112Ala or Asn71Ala showed improved activity when compared to wild-type human IL-15, supporting the hypothesis that the interaction between the residues Asn112, Asn71 with γ and β chain receptor, respectively, is critical and the molecule size on those sites interfere with IL-15 mediated γ/β chain signaling (Huang et al., 2018). In contrast, the yeast-derived IL-15 was found to stimulate the proliferation of CTLL2 cells and the human NK cells *in vitro* and *in vivo* in a SCID mouse model. In particular, *P. pastoris*-derived IL-15 showed enhanced ability to stimulate CTLL-2 cells when compared to commercially available *E. coli*-produced IL-15 (Sun et al., 2016).

To sum up, human IL-15 expressed in HEK293 cells predominantly carries glycan on Asn79 and it has been observed that glycans on this site may change IL-15 bioactivity and secretion depending on the expression system (Fig. 4). Therefore, considering its glycosylation pattern can help refine the design and expression of new therapeutic formats containing this cytokine.

### 1.15. Interleukin-17A

Interleukin-17A (IL-17A) is a proinflammatory cytokine, which binds to the IL-17 receptor as a dimer, triggering the induction of chemokine production. This leads to the recruitment of immune cells like monocytes and neutrophils to the infection or inflammation site (Fossiez et al., 1998). IL-17A is mainly produced by a subset of T cells, namely Th17 cells, and other immune cells, including NK cells, γδ T cells and innate lymphoid cells. While IL-17A has been identified as a prominent target for autoimmune disease inhibition, its angiogenic properties have limited its use in cancer treatment (Pasche et al., 2012).

Analysis of the cDNA sequence revealed a potential *N*-glycan site at Asn45 (Fig. 3A, Table 2). Upon expression in CV1/EBNA1 cells, IL-17A exhibited two different MWs bands. Treatment with tunicamycin or PNGase F eliminated the high MW band, indicating that IL-17A exists in both glycosylated and non-glycosylated form (Yao et al., 1995). HEK293-derived IL-17A, predominantly carries *N*-glycans with a bi-antennary structure terminating in GalNAc, core-fucose and 2 antenna fucoses, which is a common glycan form for proteins expressed in HEK293 cells. Some sialylated and sulfated glycan varieties were also identified (Geoghegan et al., 2013), while unexpectedly, an *O*-glycan of mucin type was identified on Thr26 (Fig. 3, Table 2) (Geoghegan et al., 2013). In contrast, *P. pastoris*-derived IL-17A resulted in a mixture of non-glycosylated and oligomannosylated species, but treatment with sialidase and *O*-glycosidase had no effect (Grinna and Tschopp, 1989). The fusion of an GSG-6xHIS-GSG linker at the cytokine N-terminus in the HEK293-derived IL-17A may have generated an artificial *O*-glycan site, suggesting that the introduction of purification/detection tags may supply sufficient amino acids to create such a site (Fossiez et al., 1996; Murphy et al., 1998). However, the functional consequences of the *O-*glycan remain unknown as no studies were performed to investigate its impact on IL-17A biological activity.

The effect of the *N-*glycan on IL17A activity was studied by measuring IL-6 secretion from human dermal fibroblasts. Purified HEK293-IL17A, containing a mixture of glycosylated and non-glycosylated IL-17A, did not show a significant difference in their activity, suggesting that glycosylation has little effect on biological activity (Geoghegan et al., 2013). However, by chemically coupling IL-17A with different glycan structures, including one GlcNAc, two GlcNAc and a fully bi-antennary glycan with sialic acid, different effects on IL-17A bioactivity were identified. The bi-antennary sialylated glycan of IL-17A showed a decreased affinity for IL-17A receptor by two-fold, as determined by surface plasmon resonance (SPR). Conversely, the presence of one GlcNAc supported *in vitro* folding and enhanced protein stability (Li et al., 2021). Despite these observations, the crystal structure obtained from HEK-cell derived IL-17A did not provide a clear indication of whether the glycans could interfere with receptor binding (Liu et al., 2013). However, IL-17A expressed in *P. pastoris* performs similarly when glycosylated or not in IL-6 secretion human dermal fibroblasts assay and forms dimers without the need for intermolecular disulfide bonds (Murphy et al., 1998).

In summary, the presence and type of *N*-glycans of IL-17A may influence its activity and binding affinity to the IL-17 receptor but does not affect dimerization, while the role and presence of an artificial *O*-glycan remains unclear (Fig. 4). This is why it is important to consider that the introduction of a N- or C-terminal fusion tag can potentially create artificial glycan sites. These findings emphasize the importance of characterizing the glycosylation patterns of IL-17A, which is crucial for ensuring their quality and efficacy in biological and therapeutic applications.

### 1.16. Interleukin-21

Interleukin-21 (IL-21) is predominantly involved in the induction of proliferation of NK cells and cytotoxic T cells and mainly produced by T effector cells. It has been described in different autoimmune diseases and is a potential target for immunosuppression with anti-IL-21 therapeutics (Chabab et al., 2020; Ren et al., 2021). Furthermore, its cancer properties have been explored, leading to the development of two immunocytokines (Maurer et al., 2012) and one fusokine (Williams et al., 2010).

IL-21 is predicted to harbor one *N*-glycan at position Asn73, but characterization of human IL-21 glycosylation status remains limited (Fig. 3A, Table 2) (Parrish-Novak et al., 2000). However, the endogenous murine IL-21 (mIL-21), is glycosylated when expressed by TS/A mouse mammary adenocarcinoma (Di Carlo et al., 2004). Notably, on an immunoblot the lower MW band (16 kDa) appeared to be higher than the *E. coli-*derived IL-21 (15 kDa), prompting further investigation into IL-21 glycosylation (Di Carlo et al., 2004). The same features were observed after coupling mIL-21 to murine GM-CSF in a fusokine format (GIFT-21) (Williams et al., 2010). Human IL-21 cDNAs, including the sequence encoding for a shorter IL-21 isoform that still contains Asn73, were expressed in HEK293T cells. After SDS-PAGE, only the canonical IL-21 isoform was larger in MW than the recombinantly expressed *E. coli* human IL-21, suggesting that human IL-21 might be glycosylated. However, further investigation is required to elucidate the difference in size between the 2 isoforms. Although glycosylation may be involved, no additional information on IL-21 glycan characterization has been published(Rahman et al., 2007).

In terms of bioactivity, there was no clear difference between *E. coli-*derived IL-21 and HEK-derived IL-21 isoforms in the ability to induce proliferation of BaF3 cell line expressing IL-21R (Rahman et al., 2007). Several other studies have shown that *E. coli-*produced IL-21 is biologically active (Lee et al., 2010) and has been tested in clinical trials (Asano et al., 2002; Davis et al., 2007), supporting the hypothesis that glycosylation is dispensable (Fig. 4).

Comprehensive glycan characterization of IL-21 is lacking, warranting further investigation to determine their involvement in secretion, receptor-binding, cytokine half-life, stability, and potential immunogenicity.

### 1.17. Interleukin-22

Interleukin-22 (IL-22) is a homodimeric cytokine that is structurally related to IL-10 but has different immune functions. While IL-10 is known to be anti-inflammatory, IL-22 fulfills a proinflammatory role in many different tissues and is involved in numerous diseases, including inflammatory bowel disease (IBD), psoriasis, and cancer, making it an interesting target or potential therapeutic. This led to the development of IL-22-Fc fusion protein which is currently explored in the treatment of IBD (Fig. 1, Table 1) (Mizoguchi et al., 2018).

The cDNA revealed three potential *N*-glycosylation sites: Asn54, Asn68, and Asn97 (Xie et al., 2000) (Fig. 3A, Table 2). When expressed in insect S2-cell IL-22 displays glycosylation across all three *N*-glycans sites, where at least one glycan site is occupied (Fig. 2A). A more elaborate glycan profiling was performed on IL-22 derived from *N. benthamiana* plant and human HEK293 cells. Plant-derived IL-22 carries a large proportion (50 %) of bi-antennary glycan with terminal Lewis A motifs (Galβ1,3(Fuc⍺1,4)GlcNAc) and β1,2-xylose, a feature commonly identified in plants. On the other hand, HEK293-derived IL-22 revealed a similar heterogenous mixture of core-fucosylated complex *N*-glycans with two or three branches, terminating either with GlcNAc, GalNAc (less), galactose and sialic acid or (sialyl)-Lewis X. When Asn54 was replaced by Ser, expressed in *N. benthamiana cells*, the total glycan profile switched towards a more paucimannosidic glycans, with a loss of Lewis A motifs, and the addition of α1,3-fucose (Fig. 2A) (Wilbers et al., 2016). This indicated that mutation Asn54 to Ser can influence the type of glycosylation placed on Asn69 and Asn97 (Wilbers et al., 2016).

To understand the role of the glycans, a crystal structure of the S2 cells-derived IL-22, including all the different glycan variants, was generated. The structural analysis suggests that Asn97 is located internally within the protein, and the initiating GlcNAc residue is participating in intramolecular hydrogen bond interactions. By contrast, the carbohydrate chains of Asn54 and Asn68 are located on the exterior of the protein, supporting their involvement in intermolecular interactions. In particular, the electron density observed for the carbohydrate residues attached to Asn54, indicates that they might contribute to homodimer formation (Jones et al., 2008; Xu et al., 2005). From the crystal structure of *E-coli*-derived IL-22, the spatial proximity of Asn54 and Asn97 to the dimerization site was verified, while Asn68 in the AB loop region was still projected towards the outside of the molecule. Thus, *N*-glycosylation may impact IL-22 dimerization, but further study is needed (Nagem et al., 2002). For its function, IL-22 engages with a receptor complex composed of IL-22R1 and IL10R2 chains, and IL-22 initially binds to IL-22R1 before interacting with IL-10R2. Binding kinetic and strength were analyzed with soluble IL-22R1 and IL-10R2 (expressed in S2 cells) in SPR. S2-derived IL-22 *vs E. coli* derived IL-22 did not show significant differences on the IL-22R1 binding kinetics and strength. However, the presence of one GlcNAc and core-fucose on Asn54 increased the binding affinity of IL-22/sIL-22R1 complex to the low-affinity IL-10R2 of approximately 8-fold (Logsdon et al., 2004). In conclusion, the glycans on IL-22 appeared to play a role in increasing binding affinity towards the IL-10R2. This was further supported by the crystal structures, suggesting that the glycan on Asn54 might induce molecular alterations in the IL-10R2 binding region (Jones et al., 2008; Xu et al., 2005).

However, this was contradicted in a following study where the glycan on Asn54 did not seem to mediate IL-22 binding to the IL-10R2. By site-directed mutagenesis of Asn54 to Gln or PNGase F treatment in *N. benthamiana* platform no significant differences in secretion and biological activity were observed. Moreover, both the glycosylated plant and HEK293 derived IL-22 species performed similarly to the *E. coli* non-glycosylated species, when tested for their ability to induce IL-10 expression in Colo-205 cells (Fig. 2A) (Wilbers et al., 2016).

Overall, even if HEK293 cell-produced IL-22 can resemble the native glycan pattern, there is not a comprehensive characterization of *N*-glycan composition on serum IL-22. The impact of glycans on IL-22 still appears to be contradictory based on the current literature (Fig. 4). Therefore, additional research is needed to truly understand the role of glycans on IL-22 and how they can be employed in the development of new IL-22 fusion constructs (WO2014145016A2).

## 2. Conclusion

Cytokines are invaluable tools in biomedical research and are components of new therapeutic designs (Fig. 1). However, their glycosylation status is often overlooked due to its complexity and heterogeneity. The type and occupancy of cytokine glycosylation are primarily determined by the expression host system used (Figs. 2A, 3B). For instance, IL-6 is not glycosylated by B cells, but can be glycosylated to different extents when derived from stimulated IL-1β monocytes and fibroblasts. Similarly, GM-CSF produced in heterologous expression platforms can display different glycosylation patterns, ranging from heavily sialylated in suspension CHO cells to non-glycosylated in *E. coli* or heavily oligomannosylated in yeast. Importantly, glycosylated cytokines can have different biological functions (Fig. 4). Glycosylation may either enhance or reduce cytokine biological activity, depending on the specific cytokine and glycosylation site. While glycosylation seems to be more beneficial for IFN-β, IFN-γ, IL-2, IL-6, and IL-22, it reduces the biological activity of IL-3, IL-4, IL-15, IL-17A and GM-CSF (Fig. 4). On the other hand, non-glycosylated forms of GM-CSF and IFN-β can be immunogenic in humans. Moreover, glycosylation on IFN-γ, GM-CSF, G-CSF IL-4, IL-6, IL-7 and IL-13 can improve their stability by inhibiting degradation by proteases and extending their half-life *in vivo*. Notably, glycosylation enhances the function of IFN-β and G-CSF by improving the solubility and preventing aggregation. Glycosylation can also support the secretion of IL-3, IL-15 and IFNγ. Overall, these properties can be harnessed to develop new cytokine fusion constructs and guide the selection of the expression platforms to obtain a desired therapeutic or immunological effect in existing and novel cytokine-based drugs based on the glycosylation profile. Additionally, it is important to underline that adding a fusion or detection tag may introduce artificial glycan sites, potentially influencing cytokine activity, as seen with IL-17A, though the real impact remains unknown.

In summary, glycosylation of cytokines can play a role in different aspects, including enhancing or reducing biological activity, immunogenicity, stability, multimerization, half-life, and secretion. Various studies have explored different host cell platforms for recombinant cytokines production, leading to the generation of distinct glycoforms with differences in biological properties, since glycosylation can be cell line, species-, tissue- and differentiation-specific. While not discussed here, cytokine receptors are also glycosylated, remarking the important role of glycosylation in the cytokine network. Also chemokines, a distinct class of cytokines, not described in this manuscript, are proteins whose properties can affect their interaction with glycosaminoglycans (GAG), leading to different immunological responses and holding potential for the design of new cytokine based therapies (*e.g.*, IL-18) (Fig. 1), only a small number of chemokines contain a glycan site, the function is often unknown or whether the glycan influence GAG binding. Lastly, systematic studies investigating the role and importance of human glycosylation of endogenous cytokines and of endogenous target receptors on target cells and tissues are largely lacking. This is due to limited protein quantities in endogenous fluids making it challenging to analyze the glycosylation patterns. However, current glycoanalytic platforms are becoming more sensitive and of higher resolution, opening new research opportunities to investigate native glycosylation of different cytokines in health and disease. Therefore, the scientific community must be aware of the implications of cytokine glycosylation when using them as new therapeutic treatments or in immunological experiments, as even slight variations in glycosylation can significantly affect the safety and effectiveness of cytokine-based therapies.

## Figures and Tables

**Table 1:** Major formats of therapeutic cytokines.

*Supercytokine*				
G-CSF	Name	Target/Features	In clinical trial: Yes/No	References
1	G-CSF tandem molecule	Locus mutation for new N-glycan site	Yes	
2	Pegfilgrastim	PEGylation	Yes	(Molineux, 2004)
3	Lipegfilgrastim	GlycoPEGylation	Yes	(Abdolzade-Bavil et al., 2013; Hoggatt et al., 2015)
4	G-CSF/IgG1-FFc	Fusion to IgG1Fc domain	No	(Cox et al., 2004)
5	G-CSF/IgG4-CH	Fusion to IgG4 CH domain	No	(Cox et al., 2004)
6	Eflapegrastim	Fusion to human IgG4-Fc fragment	FDA approval	(Molineux, 2004)
7	Balugrastim	Fusion to recombinant serum albumin	Yes	(Ghidini et al., 2016; Gladkov et al., 2016; Halpern et al., 2002; Wang et al., 2019)
8	ABD-G-CSF	Fusion to albumin binding domain	No	(Nikravesh et al., 2022)
9	3DHSA-G-CSF	Fusion to human serum albumin domain III	No	(Zhao et al., 2013)
**IFNα**				
1	Mutational IFN- 2	Locus mutation	No	(Jaitin et al., 2006)
2	IFN- mutant	Locus mutation	No	(Brideau-Andersen et al., 2007)
3	IFN- 2α mutant	Locus mutation	No	(Levin et al., 2014)
**IFNβ**				
1	IFNβ mutein, Met1, Cys17	Locus mutation	Yes	(Mark et al., 1984)
2	IFNβ mutein, R27T	Locus mutation for new N-glycan site	No	(Song et al., 2020)
3	PEG-IFNβ−1a	PEGylation	Yes	(Kieseier and Calabresi, 2012)
**IL-2**				
1	IL-2 mutein, BAY50–4798	Locus mutation (mutated sequence)	Yes	(Shanafelt et al., 2000)
2	IL-2 superkine	Locus mutation (mutated sequence)	No	(Mizui, 2019)
3	IL-2 superkine antagonist, MDNA209	Locus mutation	No	Medicenna
4	Long-acting IL-2 superkine, MDNA11	Locus mutation and fusion to albumin	No	Medicenna
5	IL-2(3×) -Fc	Locus mutation and Fc fusion	No	(Vazquez-Lombardi et al., 2017)
6	PEG-IL-2, NKTR-214, *Bempegaldesleukin*	Fusion with PEG	Yes	(Charych et al., 2016)-11
7	IL-2-mutein-Fc, AMG592	Locus mutation and fusion with Fc	Yes	(Khoryati et al., 2020)
8	IL-2 polymer, NKTR-358	Polymer	Yes	(Dixit et al., 2021)
9	IgG-(IL-2N88D)2, RG7835, *Melredableukin alfa*	Locus mutation and fusion Fc	No	(Peterson et al., 2018)
10	IL-2-mutein-PEG, THOR-707	Locus mutation and PEG	Yes	(Ptacin et al., 2021)
11	IL-2-mutein-PEG, SHR-1916,	Locus mutation and PEG	No	Hengrui
12	ALKS 4230	Fusion to IL-2Ra domain	No	
13	NARA1leukin	human IL-2 (hIL-2) antigen-binding groove of NARA1	No	(Arenas-Ramirez et al., 2016)
14	Alt-801	Fusion to single-chain TCR reactive to p53 (aa 264–272)	Yes	(Lopes et al., 2020)
**IL-4**				
1	IL-4 superkine	Locus mutation (Type-I receptor selective IL-4)	No	(Junttila et al., 2012)
2	IL-4 superkine	Locus mutation (Type-II receptor selective IL-4)	No	(Junttila et al., 2012)
**IL-7**				
1	Hyleukin-7, NeoImmuneTech	Engineered IL-7 moieties (2) hybridized with IgD or IgG4 Fc	Yes	(Kim et al., 2020)
2	hyperglycosylated IL-7, CYT107		Yes	Cyteris
**IL-10**				
1	Myeloid-Biased IL-10 variants	Locus mutation	No	(Saxton et al., 2021)
2	Affinity-enhanced IL-10 variant	Locus mutation	No	(Gorby et al., 2020)
**IL-12**				
1	IL-12 partial agonists	Locus mutation	No	(Glassman et al., 2021)
**IL-13**				
1	IL-13 superkine, MDNA413	Locus mutation	No	Medicenna
2	Modified IL-13 Superkine, fi MDNA132	Locus mutation	No	Medicenna
**IL-15**				
1	IL-15 antagonist	Locus mutation	No	(Pettit et al., 1997; Ring et al., 2012)
2	IL-15 antagonist	Locus mutation	No	(Ferrari-Lacraz et al., 2001)
3	Interleukin-15C	Fusion with IL-15 receptor to Fc	No	(Burrack et al., 2018)
4	RLI, IL-15-linker-IL-15Rα sushi domain	Fusion with sushi domain	No	(Mortier et al., 2006)
5	IL-15N72D/IL-15R -IgG1-Fc α chimera, ALT-803	Locus mutation, IL-15 receptor a-Fc fusion protein	Yes	(Rhode et al., 2016)
6	IL-15/IL-15R -IgG1-Fc complex	Fusion IL-15 receptor to IgG1-Fc complex	No	(Rubinstein et al., 2006)
**IL-18**				
1	Decoy-resistant IL-18	Locus mutation	No	(Zhou et al., 2020)
**IL-22**				
1	IL-22-IgG4Fc RG7880, UTTR1147A, *Efmarodocokin alfa*		Yes	(Wagner et al., 2023)
***Immunocytokine*** **GM-CSF**				
1	Anti-HER2/neu IgG3-(GM-CSF)	HER2/neu	No	(Dela Cruz et al., 2000; Dela Cruz et al., 2006)
2	Anti-GD2 GM-CSF	GD2	No	(Metelitsa et al., 2002)
3	CLL1-GM-CSF	MHC II	No	(Hornick et al., 1997)
4	L19- GMCSF	EDB	No	(Kaspar et al., 2007)
**IFNα**				
1	F8-INFα	EDA	No	(Frey et al., 2011)
2	CD20IgG3 IFNa	CD20	No	(Xuan et al., 2010)
3	anti-CD20-IFNα−2b	CD20	Yes	ImmunGene
4	Anti-HER2/neu-IFN	Her2/neu	No	(Huang et al., 2007)
5	C2–2b-2b	HLA-DR	No	(Rossi et al., 2011)
6	20–2b	CD20	No	(Rossi et al., 2009)
7	Nb-IFNα2 v	anti-TNFR1	No	(Garcin et al., 2014)
8	mCD20–AcTaferon	CD20	No	(Cauwels et al., 2018)
9	CD38-Attenukine^™^	CD38	No	(Pogue et al., 2016)
10	Anti-PD-L1 INFα	PD-L1	No	(Liang et al., 2018)
**IFNβ**				
1	IFNβ−1a-Her2	Her2	No	(Lee et al., 2020)
2	SL335-IFNβ−1a	Anti-serum albumin Fab	no	(Ji et al., 2019)
**IFNy**				
1	L19-IFNy	EDB	No	(Ebbinghaus et al., 2005)
2	F8-IFNγ	EDA	No	(Hemmerle and Neri, 2014a)
3	anti-PD-L1 VHH IFNy	PD-L1	No	(Dougan et al., 2018)
4	anti-CD70-IFNy	CD70	No	(Chen et al., 2013)
**TNFα**				
1	L19-TNF, *Fibromun*	EDB	Yes	(Pretto et al., 2014; Danielli et al., 2015; Menssen et al., 2018; Borsi et al., 2003; Corbellari et al., 2020)
2	F8-TNF	EDA	No	(Ziffels et al., 2018; Hemmerle et al., 2013)
3	IL-2-F8-TNFmut	EDA	No	(De Luca and Neri, 2018)
4	TNF-TNT3	DNA	No	(Sharifi et al., 2002)
5	TNF-FuP	EGFR	No	(Christ et al., 2001)
6	scFvMEL-TNF	gp240		(Liu et al., 2004; Liu et al., 2006)
7	MFE23-TNF	No	Yes	(Cooke et al., 2002)
8	ZME/TNF	gp240	No	(Rosenblum et al., 1995)
9	Anti-transferrin IgG TNF	human transferrin receptor	No	(Hoogenboom et al., 1991a; Hoogenboom et al., 1991b)
10	Anti-GD2 TNF	GD2	No	(Gillies et al., 1993)
11	Anti-FAP- TNF	FAP	No	(Bauer et al., 2009a; Bauer et al., 2004)
12	sFv23/TNF	HER-2/neu	No	(Rosenblum et al., 2000; Lyu et al., 2008)
13	TA99-mTNF	gp75	No	(Murer et al., 2019)
14	TNF-B1	LeY	No	(Scherf et al., 1996)
15	Anti-CAIX TNF	CAIX (cG250)	No	(Bauer et al., 2009b; Ziffels et al., 2019)
**IL-2**	**Name**	**Target/Features**	**Stage**	**Ref**
1	huKS-IL-2, EpCAM G733–2, EMD 273066	EpCAM	Yes	(Dolman et al., 1998)
2	Hu14.18-IL-2 EMD 273063	GD2	Yes	(Sabzevari et al., 1994)
3	NHS-IL-2LT, EMD 521873	DNA	Yes	(Gillies et al., 2011)
4	FAP-IL2v RG7461; RO6874281	FAP	Yes	(Soerensen et al., 2018)
5	DI-Leu16-IL-2	CD20	Yes	(Gillies et al., 2005)
6	CEA-IL2v, *Cergutuzumab amunaleukin*	CEA	Yes	(Xu et al., 2000)
7	L19-IL-2, *Darleukin*	EDB	Yes	(Carnemolla et al., 2002)
8	F16-IL-2, *Teleukin*	TnC A1	Yes	(Mårlind et al., 2008)
9	hT84.66/M5A-IL-2	CEA		(Sta Maria et al., 2015)
10	EGFR Ab-sumIL-2	EGFR	No	(Sun et al., 2019)
11	ch225-IL2	EGF	No	(Becker et al., 1996)
12	antiCD20- IL2	CD20	Yes	(Gillies et al., 2005)
13	Anti HER2/neu IgG3-IL2	HER2/neu	No	(Penichet et al., 2001)
14	CLL1-IL2	MHC II	No	(Hornick et al., 1997)
15	IL2-FuP	EGFR	No	(Christ et al., 2001)
16	2aG4-IL2	PS	No	(Huang et al., 2011)
17	F8 IL-2	EDA	No	(Pretto et al., 2014)
18	IL2-αCD38-αCD38-scTRAIL	CD38	No	(De Luca et al., 2018)
19	Anti-PD-L1 IL2	PD-L1	No	(Chen et al., 2016)
20	Anti-PD-L1 IL2v, *Eciskafusp Alfa*	PD-L1	Yes	(Klein et al., 2019),
21	KS-IL12/IL2	EpCAM	No	
22	anti-PD-L1 VHH IL-2	PD-L1	No	(Dougan et al., 2018)
23	Anti-CAIX IL2	CAIX (cG250)	No	(Ziffels et al., 2019)
24	2C9-IgG1-IL-2, KM281	PSMA	No	(Sugimoto et al., 2014)
25	FUMK1- IL2	MK1	No	(Matsumoto et al., 2002)
26	IL2-MOV19	aFR	No	(Melani et al., 1998)
**IL-4**				
1	F8-IL4	EDA	No	(Hemmerle and Neri, 2014b)
**IL-7**				
1	F8-IL7	EDA	No	(Pasche et al., 2011)
2	F8-IL7-F8	EDA	No	(Pasche et al., 2011)
**IL-9**				
1	F8-IL19	EDA	No	(Heiss et al., 2022)
**IL-10**				
1	F8-IL10, *Dekavil*	EDA	No	(Schwager et al., 2009)
**IL-12**				
1	NHS-IL-12, M9241, MSB0010360	DNA	Yes	(Fallon et al., 2014)
2	NHS-muIL12	DNA	No	(Xu et al., 2017)
3	HuBC1-IL-12, AS1409	EDB	Yes	(Lo et al., 2007)
4	L19-mIL12	EDB	Yes	(Weiss et al., 2020)
5	chTNT3-IL12	DNA	No	(Li et al., 2004)
6	KS-IL12	EpCAM	No	(Gillies et al., 1998)
7	KS-IL12/IL2	EpCAM	No	(Gillies et al., 2002)
8	mscIL-12 her2.IgG3	HER2/neu	No	(Peng et al., 1999)
9	IL12-scFv-L19	EDB	No	(Halin et al., 2002)
10	IL-12-SIP (L19)	EDB	No	(Gafner et al., 2006)
11	L19p35/p40L19	EDB	No	(Gafner et al., 2006)
12	F8p35/p40F8 (F8-IL12)	EDA	No	(Sommavilla et al., 2010)
**IL-13**				
1	F8-mIL13	EDA	No	(Hess and Neri, 2015)
**IL-15**				
1	PD-L1-IL-15	PD-L1	No	(Knudson et al., 2020)
2	RGD-IL-15	integrin	Yes	(Chen et al., 2015; Spitzer et al., 2017)
3	L19-IL15	EDB	No	(Kaspar et al., 2007)
4	scFv36_RD_IL-15	FAP	No	(Kermer et al., 2012)
5	Anti-GD2-RLI	GD2	No	(Vincent et al., 2013)
6	Anti-CD20-RLI	CD20	No	(Vincent et al., 2014)
**IL-17**				
1	F8-IL17	EDA	No	(Pasche et al., 2012)
**IL-21**				
1	EGFR-IL-21	EGFR	No	(Deng et al., 2020)
2	PD-1-IL-21	PD-1	No	(Shen et al., 2020)
***Engager cytokines***				
**IL-15**	**Name**	**Target/Features**	**Stage**	**References**
1	CD16-IL-15-CD3, OXS-3550	CD3 and CD16	Yes	(Schmohl et al., 2017)
2	CD16-IL-15-CD19	CD19 and CD19	No	(Cheng et al., 2020)
***Fusokine***				
**GM-CSF**	**Name**	**Target/Features**	**Stage**	**References**
1	GM CSF(N-terminal) CCL2 (GMME1)	Fusion of GM-CSF and CCL2	No	(Rafei et al., 2009)
2	GIFT2 (GM CSF-IL-2)	Fusion of GM-CSF and IL-2	No	(Penafuerte et al., 2009)
3	GIFT4 (GM CSF-IL-4)	Fusion of GM-CSF and IL-4	No	(Deng et al., 2014)
4	GIFT7 (GM CSF-IL-7)	Fusion of GM-CSF and IL-7	No	(Shireman et al., 2023)
5	mSCH-GM-CSF/mSCH-IL-2	Dual cytokine heterominibodies with murine GM-CSF and IL-2	No	(Schanzer et al., 2006)
**IFN-β**				
1	IFNβ-Gal-9	Galectin-9	No	(Hamana et al., 2018)
**TNFα**				
1	NGR-hTNF	Fusion of peptide CNGRCG and TNFα	Yes	(Gregorc et al., 2010)
**IL-2**				
1	GIFT2 (GM CSF-IL-2)	Fusion of GM-CSF and IL-2	No	(Penafuerte et al., 2009)
2	mSCH-GM-CSF/mSCH-IL-2	Dual cytokine heterominibodies with murine GM-CSF and IL-2	No	(Schanzer et al., 2006)
**IL-3**				
1	IL-3-GM-CSF (PIXY123)		No	(Curtis et al., 1991; O’Shaughnessy et al., 1996; Vadhan-Raj et al., 1994)
2	DT388IL3 (Tagraxofusp)	Diphtheria toxin-IL-3	Approved	(Syed, 2019)
**IL-4**				
1	GIFT4 (GM–CSF-IL-4)	Fusion of GM-CSF and IL-4	No	(Deng et al., 2014)
2	MDNA55	Fusion of an engineered circularly permuted Interleukin-4 and exotoxin A of *Pseudomonas aeruginosa*	No	(Joshi et al., 2001)
3	IL4–10	Fusion of IL-4 and IL-10	No	(Steen-Louws et al., 2019a)
**IL-6**				
1	IC7Fc: IL-6, domain of CNTF, Fc	IL-6 and CNTF-based synthekines	No	(Findeisen et al., 2019)
**IL-7**				
1	GIFT7 (GM CSF-IL-7)	Fusion of GM-CSF and IL-7	No	(Shireman et al., 2023)
**IL-10**				
1	IL4–10	Fusion of IL-4 and IL-10	No	(Steen-Louws et al., 2019b)
**IL-15**				
1	GIFT15	Fusion of GM-CSF and IL-15	No	(Rafei et al., 2007)
**IL-21**				
1	GIFT21 (GM–CSF-IL-21)	Fusion of GM-CSF and IL-21	No	(Williams et al., 2010)

**Table 2:** Cytokine length, size, occupancy of the glycans sites, expression platforms and multimerization.

Cytokine	Form	Amino acid (aa) length	Signal peptide (SP)	Glycosylation sites	Occupancy*	Expression system	Molecular Weight	Uniprot no
GM-CSF	Monomer	144	1–17	Ser5, Ser7, Ser9, Thr10, Asn27, Asn37	Ser5 (low), Ser7 (100%), Ser9, Thr10, Asn27 (60%), Asn37 (85%)	*E.coli*, COS-7, CHO, Namalwa, PHA-stimulated T cells	16.3–35 kDa, 20–35 kDa, 20–25 kDa, 16–18 kDa	P04141
G-CSF	Monomer	177	1–30	Thr136^a^	Thr136	*E.coli*, *S. cerevisiae*, *P. pastoris*, *N. benthamiana*, CHO, CHU-2	19 kDa	P09919
IFN-β	Monomer	187	1–21	Asn80	Asn80 (75–100 %)	*E.coli*, *S. cerevisiae*, *P. pastoris* Immune Complex (IC) stimulated primary human fibroblasts, murine MG-63 cells, CHO, PC8, C127, FS-4 fibroblasts, synthesis	22–25 kDa, 18 kDa	P01574
IFN-y	Monomer/Homodimer	166	1–23	Asn25, Asn97	Asn25, Asn97	*E.coli*, PBMCs, HBL-38, COS-7, Jurkat, NK92MI, CHO	19.3–25 kDa, 20 kDa, 25 kDa	P01579
TNF-α	Homotrimeric	233	1–35	Ser4^b^	Ser4	BALL-1, HEK, HepG2	17 kDa, 26 kDa	P01375
IL-2	Monomer	153	1–20	Thr3	Thr3 (40–90 %)	*E.coli*, BHK, Ltk, CHO, PHA-stimulated T cells, Daudi, humantonsil, PBMCs, Jurkat	14 kDa, 16kDa-17kDa	P60568
IL-3	Monomer	152	1–19	Asn15, Asn70	Asn15 (100 %) Asn70 (35 %)	*E.coli*, *P. pastoris*, High-Five, Sf9, COS-1	22–25 kDa, 19 kDa, 15 kDa	P08700
IL-4	Monomer	153	1–24	Asn38, Asn105	Asn38	*S. Cerevisiae*, *P. pastoris*, CI27, HEK293T, CHO	15 kDa, 45–95 kDa	P05112
IL-6	Mono, Homodimer and Oligomers	212	1–27/29^cc^	Asn46, Asn145, Thr139	Asn46	*E.coli*, IL-1β-stimulated monocytes, PBMCs, human fibroblast cells, CHO, COS-7, PA317, GH3, sfBJAB, RPMI788, TM-1, HS-2, semi chemically synthesized	21,5 kDa, 23,5 kDa, 24 kDa, 26 kDa, 28 kDa	P05231 (Q75MH2)
IL-7	Monomer	177	1–25	Asn70, Asn91, Asn116, Thr111	Asn70, Asn91, Asn116, Thr111	*E.coli*, *P. pastoris*, Sf9, Sf21, CHO-K1, CHO, HEK293T, COS-7	17 kDa, 18 kDa, 21kDa, 25 kDa, 33.5 kDa, 40–80 kDa	P13232
IL-9	Monomer	144	1–18	Asn32, Asn45, Asn60, Asn96	unknown	Sf9, CHO	14 kDa, 23 kDa	P15248
IL-12α	Heterodimer with β	219	1–57 1–22^d^	Asn71, Asn85	Asn71 Asn85	CHO, RPMI 8866, HEK293T, COS-7	27 kDa, 30 kDa, 31 kDa, 35 kDa	P29459
IL-12β	Heterodimer with α	328	1–22	Asn103, Asn113, Asn200, Asn281, Trp297, Trp300	Asn103, Asn113 (low), Asn200 (high), Asn281 (low), Trp297 (high), Trp300 (high)	CHO, RPMI 8866, HEK293T, COS-7	39.5 kDa, 41.5 kDa	P29460
IL-13	Monomer	146	1–24	Asn28, Asn39, Asn47, Asn62	Asn28, Asn47, Asn62	*E. coli*, *Nicotiana tabacum cv.*, B9, NS0, HEK293, COS-7	10 kDa, 17 kDa	P35225
IL-15	Monomer	162	1–29	Asn90, Asn98, Asn131	Asn28	*E. coli P. pastoris*, CHO, HEK293, CV1-EBNA1	14–15 kDa	P40933
IL17A	Monomer	155	1–23	Asn45	Asn45, Thr26^e^	HEK293, CV1-EBNA-1, chemically synthesized	30 kDa	Q16552
IL-21	Monomer	162	1–24	Asn73	unknown	15 kDa	*E. coli,* CHO, HEK293	Q9HBE4
IL-22	Monomer	179	1–33	Asn21, Asn35, Asn64	Asn21, Asn35, Asn64	*E. coli*, *N. benthamiana*, S2, HEK293	unknown	Q9GZX6

* Occupancy confirms the presence of glycosylation at predicted sites, verified through SDS-page or mass-spectrometry. When available, additional information on the occupation level is reported as % or described as high, low. Glycosylation sites are always reported without the SP.

a Natural human G-CSF exists in two forms, a 177 (isoform A) and 174 (isoform B) amino-acid long protein. The glycosylation site reported refers to isoform A, where the O-glycosylation site was firstly verified.

b TNF-α precursor consists of 233 amino acids, including the SP. The mature TNF-α is cleaved after the leader peptide (76 aa) with the glycosylation site located at Ser80 (Ser4 after cleavage).

c IL-6 exists in two different N-terminal isoforms that undergo different SP cleavage. Glycosylation sites refer to the amino acid sequence without 27-amino acid long SP.

d Two distinct isoforms of IL-12α exist: one with a longer SP (57 aa) and one with a shorter SP (22 aa) which is part of the longer isoform. The reported glycosylation sites refer to IL-12α without the short SP.

e IL-17A might contain an additional O-glycosylation site due to purification tags fused at the N-terminus.
